# Functional diversity of *NLRP3* gain-of-function mutants associated with CAPS autoinflammation

**DOI:** 10.1084/jem.20231200

**Published:** 2024-03-26

**Authors:** Camille Cosson, Romane Riou, Danish Patoli, Tingting Niu, Amaury Rey, Marine Groslambert, Charlotte De Rosny, Elodie Chatre, Omran Allatif, Thomas Henry, Fabienne Venet, Florian Milhavet, Guilaine Boursier, Alexandre Belot, Yvan Jamilloux, Etienne Merlin, Agnès Duquesne, Gilles Grateau, Léa Savey, Alexandre Thibault Jacques Maria, Anne Pagnier, Solène Poutrel, Olivier Lambotte, Coralie Mallebranche, Samuel Ardois, Olivier Richer, Irène Lemelle, Frédéric Rieux-Laucat, Brigitte Bader-Meunier, Zahir Amoura, Isabelle Melki, Laurence Cuisset, Isabelle Touitou, Matthias Geyer, Sophie Georgin-Lavialle, Bénédicte F. Py

**Affiliations:** 1 CIRI, Centre International de Recherche en Infectiologie, Univ Lyon, Inserm, U1111, Université Claude Bernard Lyon 1, CNRS, UMR5308, ENS de Lyon, Lyon, France; 2 Tongji University Cancer Center, Shanghai Tenth People’s Hospital, School of Medicine, Tongji University, Shanghai, China; 3 Univ Lyon, ENS de Lyon, Inserm, CNRS SFR Biosciences US8 UAR3444, Université Claude Bernard Lyon 1, Lyon, France; 4 Institute for Regenerative Medicine and Biotherapy, Inserm, U1183, University of Montpellier, Montpellier, France; 5 Department of Molecular Genetics, Medical Genetics of Rare and Autoinflammatory Disease Unit, Montpellier University Hospital, Montpellier, France; 6 Centre de Référence des Maladies Autoinflammatoires et des Amyloses Inflammatoires, CEREMAIA, France; 7 Pediatric Nephrology, Rheumatology, Dermatology Department, National Referee Centre for Rheumatic and Autoimmune Diseases in Children (RAISE), Hôpital Femme-Mère-Enfant, Hospices Civils de Lyon, Bron, France; 8 Lyon Immunopathology Federation (LIFE), Université Claude Bernard Lyon 1, Lyon, France; 9 Service de Médecine Interne, Hôpital de la Croix-Rousse, Hospices Civils de Lyon, Lyon, France; 10 Department of Pediatrics, Clermont-Ferrand University Hospital, Clermont-Ferrand, France; 11 Sorbonne Université, Department of Internal Medicine, National Reference Center for Autoinflammatory Diseases and AA Amyloidosis, Tenon Hospital, Assistance Publique-Hôpitaux de Paris, Paris, France; 12 Internal Medicine and Onco-Immunology (MedI^2^O), Institute for Regenerative Medicine and Biotherapy (IRMB), Saint Eloi Hospital, Montpellier University, Montpellier, France; 13 Centre Hospitalier Universitaire Grenoble Alpes, Immunologie Clinique, Immuno-Hémato-Oncologie (IHO), Hôpital Couple-Enfant, Grenoble, France; 14 Service de Médecine Interne, Hospices Civils de Lyon, Edouard Herriot Hospital, Lyon, France; 15 Assistance Publique-Hôpitaux de Paris, Service de Médecine Interne et Immunologie Clinique, Groupe Hospitalier Universitaire Paris Saclay, Hôpital Bicêtre, Le Kremlin-Bicêtre, France; 16 Université Paris Saclay, Inserm UMR, 1184, CEA, Le Kremlin-Bicêtre, France; 17 Université d’Angers, Université de Nantes, Inserm, CNRS, CRCI2NA, SFR ICAT, Angers, France; 18 Médecine Interne et Immunologie Clinique, Centre Hospitalier Universitaire de Rennes, Rennes, France; 19 Paediatric, Rheumatology and Paediatric Internal Medicine, Reference Center for Rheumatic, Autoimmune and Systemic Diseases in Children (RAISE), Children’s Hospital, Bordeaux, France; 20 Department of Pediatric Onco-hematology, Children Hospital, University Hospital of Nancy, Lorraine University, Vandoeuvre-lès-Nancy, France; 21 Université Paris Cité, Institut Imagine, Laboratory of Immunogenetics of Pediatric Autoimmune Diseases, Inserm UMR 1163, Paris, France; 22 Pediatric Immunology, Hematology and Rheumatology Department, Hôpital Necker, Assistance Publique-Hôpitaux de Paris (AP-HP), Paris, France; 23 Assistance Publique-Hôpitaux de Paris (AP-HP)-Sorbonne Université, Hopital Pitié-Salpétrière, Institut E3M, Service de Médecine Interne 2, Centre National de Référence Lupus et Syndrome des Anticorps Antiphospholipides, Centre d’Immunologie et des Maladies Infectieuses (CIMI), Paris, France; 24 General Pediatrics, Infectious Disease and Internal Medicine Department, Hôpital Robert Debre, Assistance Publique-Hôpitaux de Paris (AP-HP), Reference Center for Rheumatic, AutoImmune and Systemic Diseases in Children (RAISE), Paris, France; 25 Laboratory of Neurogenetics and Neuroinflammation, Imagine Institute, Paris, France; 26 Université Paris Cité, Service de Médecine Génomique des Maladies de Système et D’Organe, Hôpital Cochin, Assistance Publique-Hôpitaux de Paris, Paris, France; 27 Institute of Structural Biology, University of Bonn, Bonn, Germany; 28 Centre Hospitalier Universitaire d'Angers, Pediatric Immuno-Hemato-Oncology Unit, France

## Abstract

*NLRP3*-associated autoinflammatory disease is a heterogenous group of monogenic conditions caused by *NLRP3* gain-of-function mutations. The poor functional characterization of most *NLRP3* variants hinders diagnosis despite efficient anti-IL-1 treatments. Additionally, while NLRP3 is controlled by priming and activation signals, gain-of-functions have only been investigated in response to priming. Here, we characterize 34 *NLRP3* variants in vitro, evaluating their activity upon induction, priming, and/or activation signals, and their sensitivity to four inhibitors. We highlight the functional diversity of the gain-of-function mutants and describe four groups based on the signals governing their activation, correlating partly with the symptom severity. We identify a new group of *NLRP3* mutants responding to the activation signal without priming, associated with frequent misdiagnoses. Our results identify key NLRP3 residues controlling inflammasome activity and sensitivity to inhibitors, and antagonistic mechanisms with broader efficacy for therapeutic strategies. They provide new insights into NLRP3 activation, an explanatory mechanism for NLRP3-AID heterogeneity, and original tools for NLRP3-AID diagnosis and drug development.

## Teaser

Functional characterization of 34 NLRP3 variants identifies benign polymorphisms versus a spectrum of gain-of-function pathogenic variants with variable effects and highlights diversities in the signals controlling their activation and sensitivity to inhibitors. This study provides tools for CAPS diagnosis and anti-inflammation drug development and insights on NLRP3 control mechanisms.

## Introduction

Cryopyrinopathies (cryopyrin-associated periodic syndrome, CAPS), now referred to as *NLRP3*-associated autoinflammatory diseases (*NLRP3*-AID), were historically described as three dominant clinical entities: familial cold urticaria (FCAS), Muckle–Wells syndrome (MWS), and neonatal-onset multisystem inflammatory disease (NOMID, also referred to as chronic infantile neurological cutaneous and joint syndrome, CINCA) (Hoffman et al., 2001; Booshehri and Hoffman, 2019). All subtypes classically display cold-induced urticaria. FCAS was described as a benign form with mainly cutaneous features and arthralgia, whereas MWS patients were described with urticaria, chronic inflammation, and even recurrent fever, sensorineural deafness, ocular inflammation, headache, arthritis, and could be complicated by inflammatory AA amyloidosis. NOMID begins at birth and is characterized by central nervous system inflammation such as chronic meningitis, skin involvement with a diffuse non-itchy urticarial rash, joint involvement including deforming arthropathy preferentially affecting the knees and facial dysmorphia characterized by the presence of frontal bumps and nasal saddle deformation. NLRP3-AID is now rather considered as a spectrum of autoinflammatory diseases as patients may experience various symptoms and cannot always be classified into the three main historical phenotypes. AA amyloidosis can complicate all forms of NLRP3-AID.

Most NLRP3-AID patients are highly responsive to IL-1–targeted therapies, especially on cutaneous, ocular, and articular features, and prevention of inflammatory amyloidosis (Booshehri and Hoffman, 2019). Nevertheless, the clinical response in patients with stable deafness, bone deformity, and chronic renal failure is often poor, which is why early diagnosis is critical to prevent irrevocable damage. Besides clinical presentation, NLRP3-AID diagnosis mostly relies on genetics. However, classical genetic approaches, including analysis of familial segregation and recurrent association with the disease, are poorly efficient to distinguish the gain-of-function pathogenic *NLRP3* mutations from non-pathogenic variants. Indeed, NLRP3-AID is a rare disease whose prevalence ranges between one and three per million. Despite some genotype–phenotype correlations, the symptoms and the severity may be variable between patients bearing identical mutations, even within one family. In addition, few low penetrance variants of *NLRP3* are associated with typical or atypical NLRP3-AID phenotypes but are also found in asymptomatic people. Finally, NLRP3-AID can be caused by somatic mosaicism, especially in NOMID often associated with de novo mutations (Louvrier et al., 2020). Of the 204 amino acid substitutions or deletions in *NLRP3* described today, only 11% have been fully determined to be pathogenic (*n* = 22) or benign (*n* = 1), while 49% are likely pathogenic (*n* = 96) or likely benign (*n* = 4), and 40% are variants of uncertain significance (*n* = 51), unsolved (*n* = 6), or not classified (*n* = 24) (Van Gijn et al., 2018).

NLRP3 is a cytosolic stress sensor that upon activation assembles the inflammasome signaling complex controlling IL-1β and IL-18 secretion and pyroptosis, a proinflammatory cell death coupled with the release of many alarmins. NLRP3 is activated by a two-step mechanism engaged by coordinated priming and activation signals. Priming is usually provided by pattern recognition receptor ligands, including the Toll-like receptor 4 agonist LPS, as well as cytokines. Priming was first described to increase the cellular level of key proteins in the pathway by transcriptional upregulation of *NLRP3* itself, the inducible inflammasome substrate pro-IL-1β and many other inflammatory genes (Bauernfeind et al., 2009). Nevertheless, priming is now recognized to also trigger an ensemble of NLRP3 posttranslational modifications that renders NLRP3 competent for activation (Juliana et al., 2012; McKee and Coll, 2020; Niu et al., 2021). Activation signals correspond to various cell stresses, most of which target plasma membrane ion permeability (including the bacterial toxin nigericin), lysosomal rupture, or mitochondrial functions. These signals converge toward a disruption of vesicular trafficking, which could be the common trigger for NLRP3 activation (Zhang et al., 2023). Then, NLRP3 assembles an inflammasome complex controlling caspase-1 activation. Caspase-1 cleaves pro-IL-1β and pro-IL-18 into their mature forms, as well as Gasdermin D (GSDMD), which in turn forms pores in the plasma membrane and ultimately mediates IL-1β/18 release and initiates pyroptosis.

Due to the aforementioned limitations of classical genetic approaches, the identification of gain-of-function *NLRP3* variants requires functional approaches. In addition, the diversity of symptoms and their genotype correlation suggests that gain-of-function mutants may be heterogeneous in their activity and that clinical manifestations may depend, at least in part, on the grade of gain-of-function. Current functional characterizations of *NLRP3* variants are largely based on the detection of IL-1β secretion in response to priming by LPS (Booshehri and Hoffman, 2019; Fayand et al., 2023). Nevertheless, this approach does not distinguish between constitutively active and priming-activated mutants and does not detect mutations that would bypass the priming requirement.

We have developed a functional cell-based assay to screen for *NLRP3* variants that uncouples NLRP3 induction, priming, and activation. For 34 *NLRP3* variants, we assessed pyroptosis and IL-1β/18 secretion from *NLRP3*-deficient U937 cells reconstituted with doxycycline-inducible NLRP3 variants in response to NLRP3 induction, priming, and/or activation. The results were confirmed in primary monocytes from 22 patients carrying 11 different variants. These analyses efficiently discriminated gain-of-function mutants from polymorphisms without any impact on NLRP3 activity and highlighted the heterogeneity of the gain-of-function mutants that partly correlated with the severity of the symptoms. In particular, we identified a group of mutants responsive to the activation signal in the absence of prior priming that could not be detected in previous functional assays, leading to misdiagnosis of non-NLRP3-AID. Moreover, our study identified some key residues in the control of NLRP3 activity and sensitivity to inhibitors, as well as inhibitor mechanisms that are efficient toward the widest range of *NLRP3* mutants.

## Results

### Reconstitution of *NLRP3*-deficient U937 human monocytes with inducible *NLRP3* variants associated with autoinflammation

**Figure 1.**
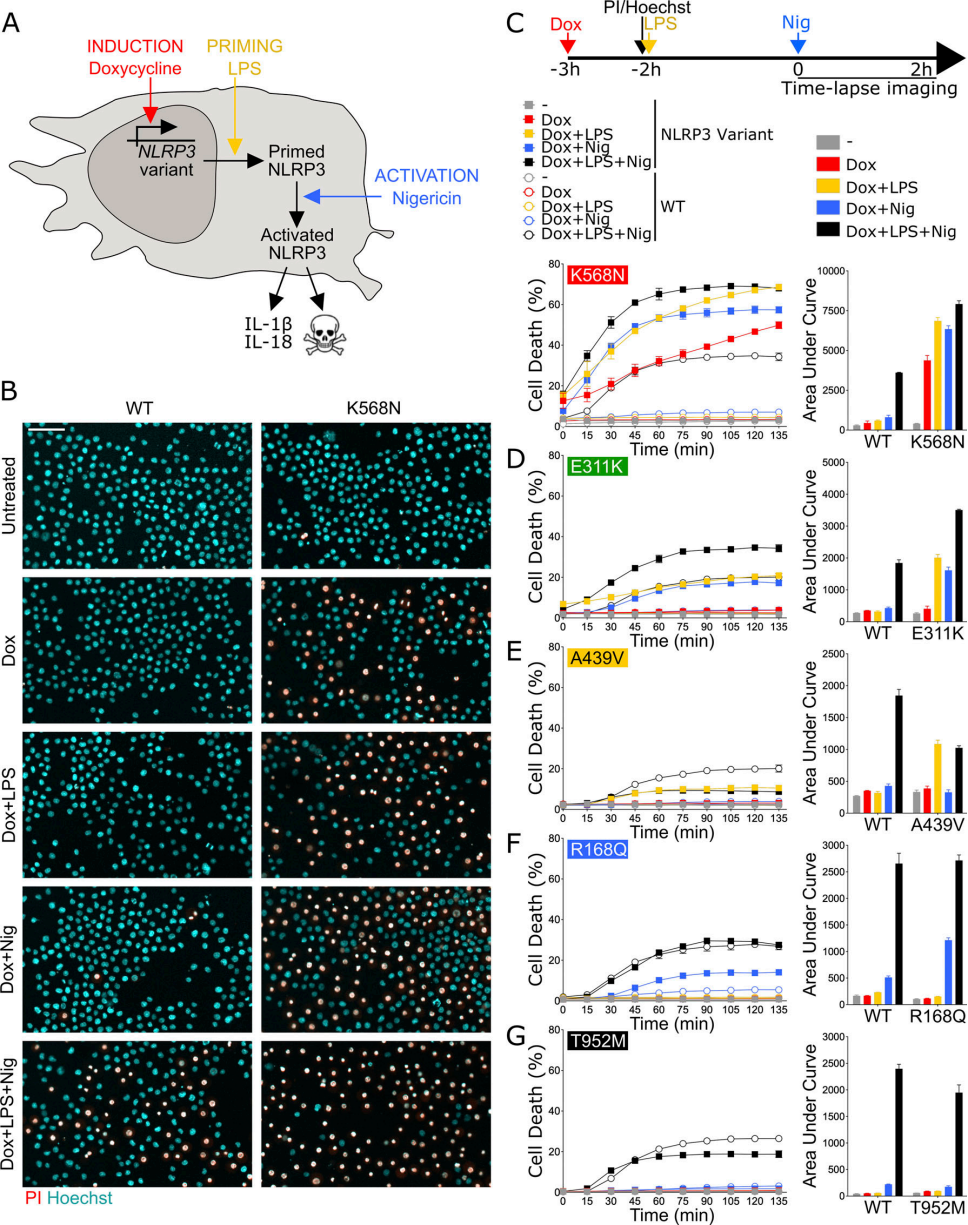
**NLRP3 expression, priming, and/or activation triggers pyroptosis in reconstituted U937 depending on the *NLRP3* variants. (A–G)** *NLRP3*-deficient U937 cells reconstituted with doxycycline-inducible *NLRP3* variants were treated with doxycycline (1 μg/ml, 3 h), LPS (40 ng/ml, 2 h), and nigericin (15 μg/ml) before cell death was monitored by PI incorporation over time quantified by time-lapse high content microscopy. **(B)** Example of microscopy images for WT and K568N (60 min, objective 10×, scale bar 100 μm). **(C)** K568N (group#5). **(D)** E311K (group#4). **(E)** A439V (group#3). **(F)** R168Q (group#2). **(G)** T952M (group#1). Means of duplicates and 1 SD (left panel) and means of AUC of duplicates and 1 SD (right panel) for cells expressing NLRP3 variants (full square) and WT (open circle) are represented. One experiment done in duplicates representative of two to eight independent experiments is shown (as indicated for each variant on the right side). Results obtained with one variant typical of each functional group are represented. Results obtained with all tested variants are presented in Fig. S2, B–F. Statistical analysis including all independent experiments are represented in Fig. 2.

**Figure S1.**
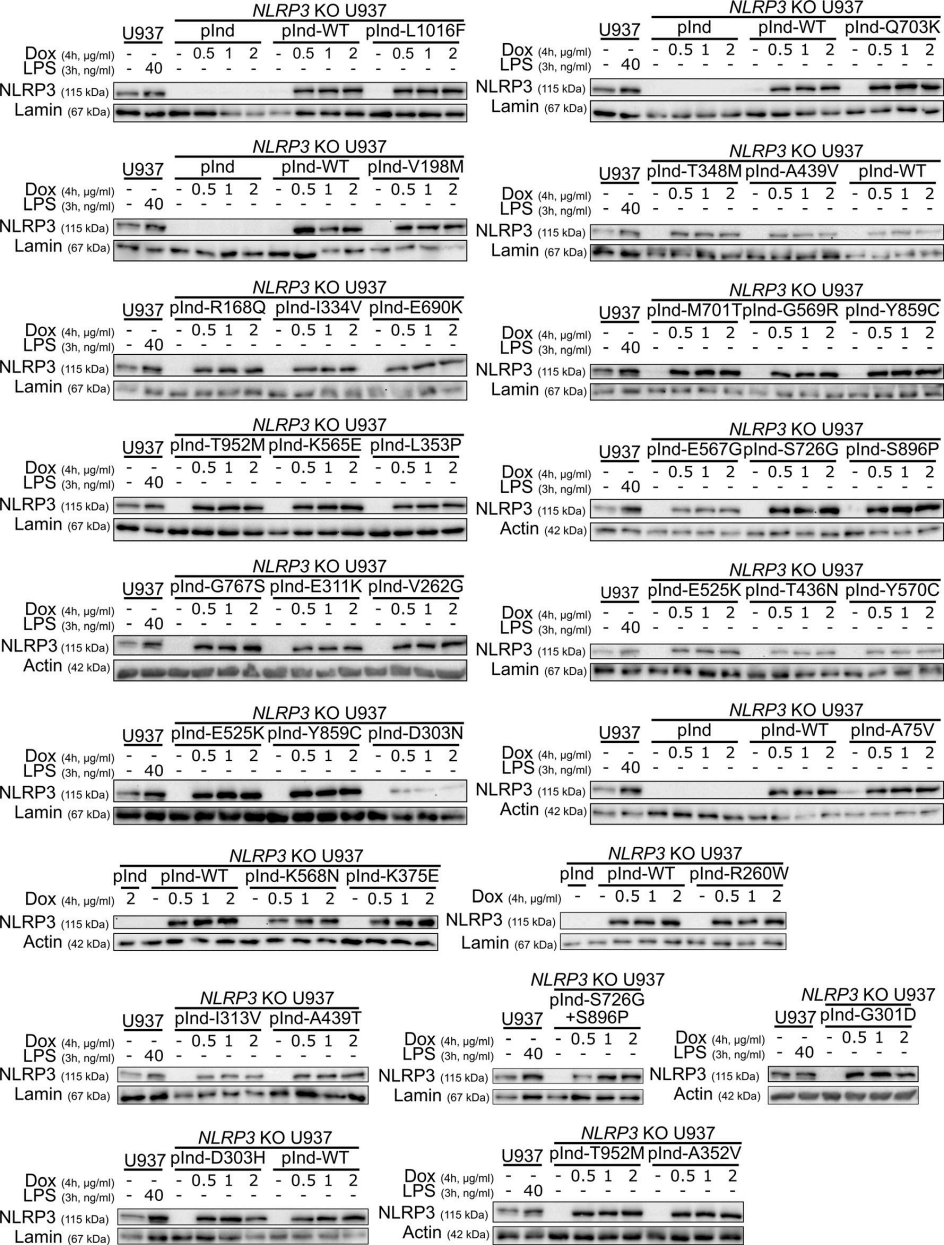
**Reconstitution of *NLRP3*-deficient U937 with doxycycline-inducible *NLRP3* variants.** *NLRP3*-deficient U937 reconstituted with doxycycline-inducible WT NLRP3 (pInd-WT), indicated *NLRP3* variants, and the empty vector (pInd) were treated with doxycycline (indicated doses, 4 h). U937 treated with LPS (40 ng/ml, 3 h) was used as a control. NLRP3 protein levels were analyzed by WB. Source data are available for this figure: SourceData FS1.

In this study, we analyzed 34 *NLRP3* variants identified in patients with symptoms suggestive of NLRP3-AID (Table 1). First, 23 variants were selected among the most common variants associated with NLRP3-AID in France (Cuisset et al., 2011), and 11 variants were included along the study upon the request of clinicians for diagnostic purposes. To compare the impact of *NLRP3* variants on inflammasome activity in a standardized genetic background, we used U937 human monocytes knocked-out for *NLRP3* by CRISPR/Cas9 and stably reconstituted them with doxycycline-inducible *NLRP3* variants (Fig. 1 A) (Lagrange et al., 2018). The expression levels of *NLRP3* variants were similar to or lower than the level of control WT *NLRP3* in reconstituted U937 cell lines treated with doxycycline, excluding that their putative gain-of-function may be caused by higher expression levels (Fig. S1). In addition, NLRP3 levels in doxycycline-treated reconstituted U937 monocytes were similar to endogenous NLRP3 levels in U937 cells treated with LPS, indicating that the system was physiologically relevant (Fig. S1).

**Table 1.** *NLRP3* variants included in the study and summary of the analysis (Infevers and GnomAD v4.0.0 databases, personal observations, and Louvrier et al. [2020])

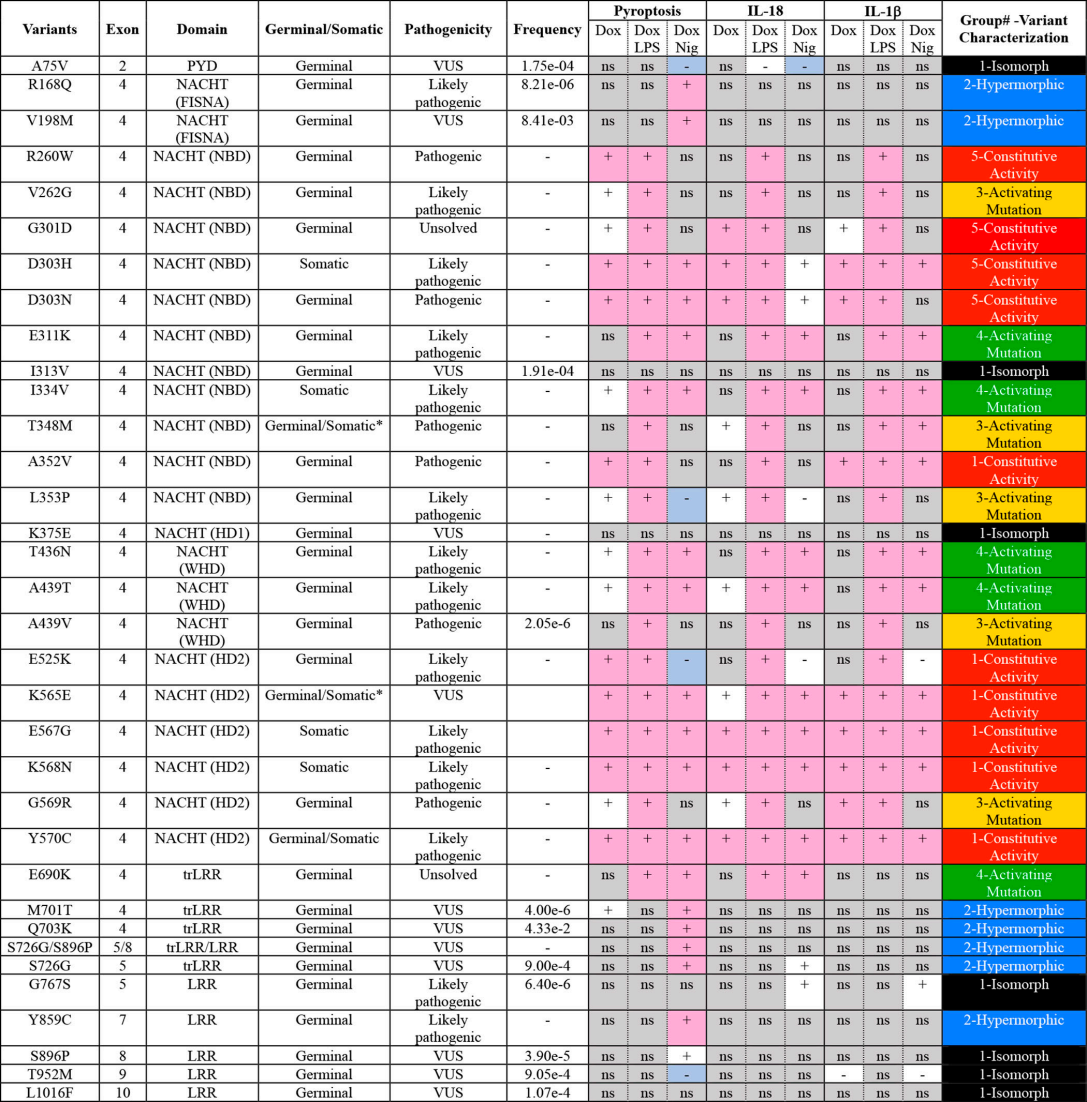

**Figure 2.**
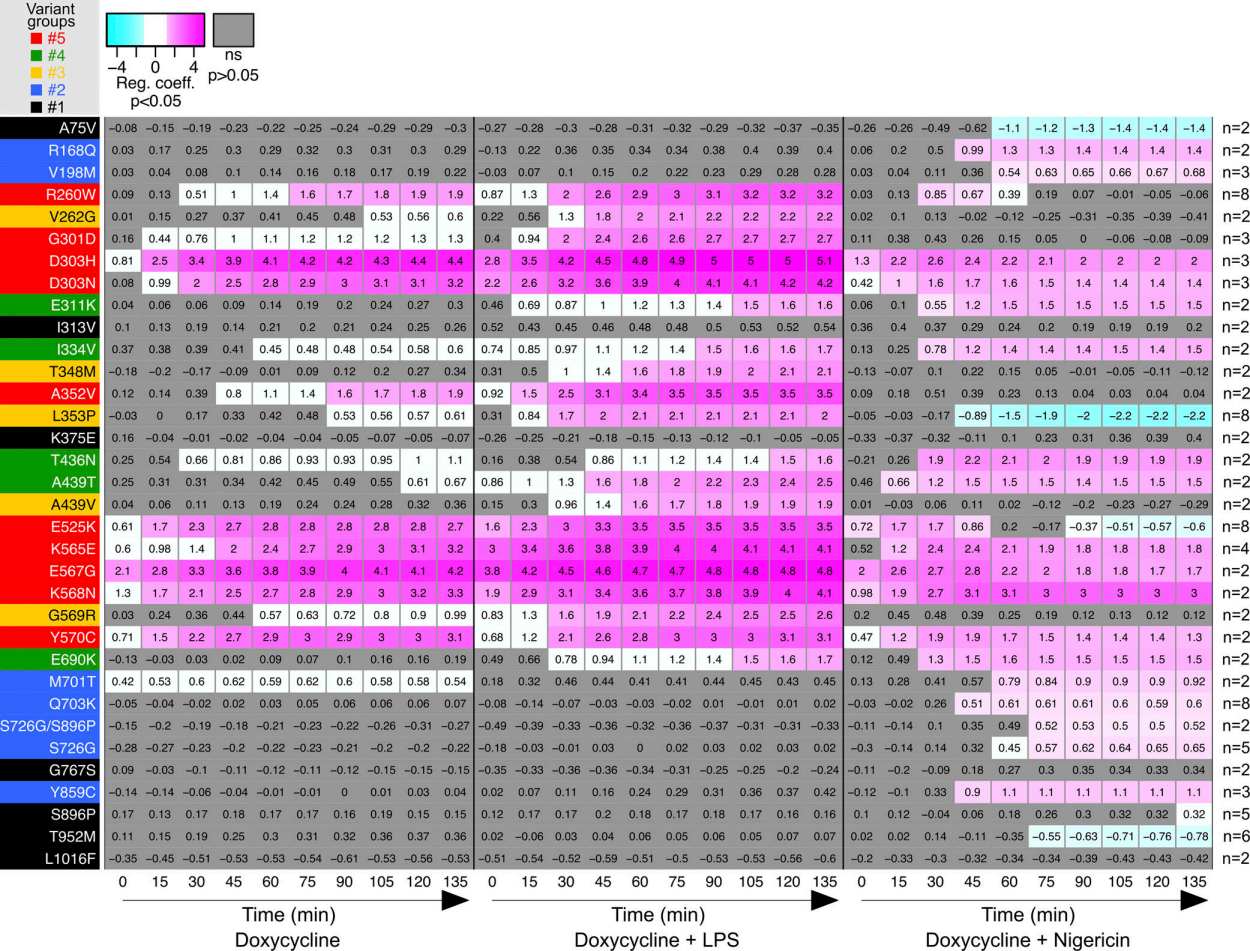
**Relative cell death of U937 expressing *NLRP3* variants as compared with U937 expressing WT *NLRP3* in response to NLRP3 expression, priming, and/or activation.** Regression coefficient (RC) heatmap from the glmm for each variant as compared with the WT. Positive RC denotes increased cell death in the considered variant as compared with the WT, and conversely. For significant values (P < 0.05), RC corresponding to increases (magenta) or decreases (cyan) in cell death are color-coded. In gray, not significant values (P > 0.05) correspond to conditions (variant * treatment * time point) in which the percentage of dead cells among U937 expressing NLRP3 variant is not statistically different from those among U937 expressing WT NLRP3. Based on the results, variants are classified into five functional groups: constitutive active variants (group#5, red), variants active upon either priming or activation signal (group#4, green), variants active upon priming signal (group#3, yellow), variants active upon activation signal (group#2, blue), and mutants active upon priming and activation signals (no gain-of-function, group#1, black). Results of two to eight independent experiments done in duplicates.

Frequencies based on exome sequencing of the general population of all origins. *, patients with somatic mutation were asymptomatic. For each variant, results obtained in U937 cells are summarized (see Figs. 2 and S4). For pyroptosis, the summary is based on PI incorporation at the time point 135 min. ns, non significant in gray; +, increased compared with WT (pink if above fixed threshold); −, decreased compared with WT (blue if below fixed threshold). In the right column, variant characterizations are color-coded according to their respective group described in Fig. 2 (group#5 in red, #4 in green, #3 in yellow, #2 in blue, #1 in black). PYD, pyrin domain; VUS, variants of uncertain significance.

### Functional screen of *NLRP3* variants using pyroptosis as readout

**Figure S2.**
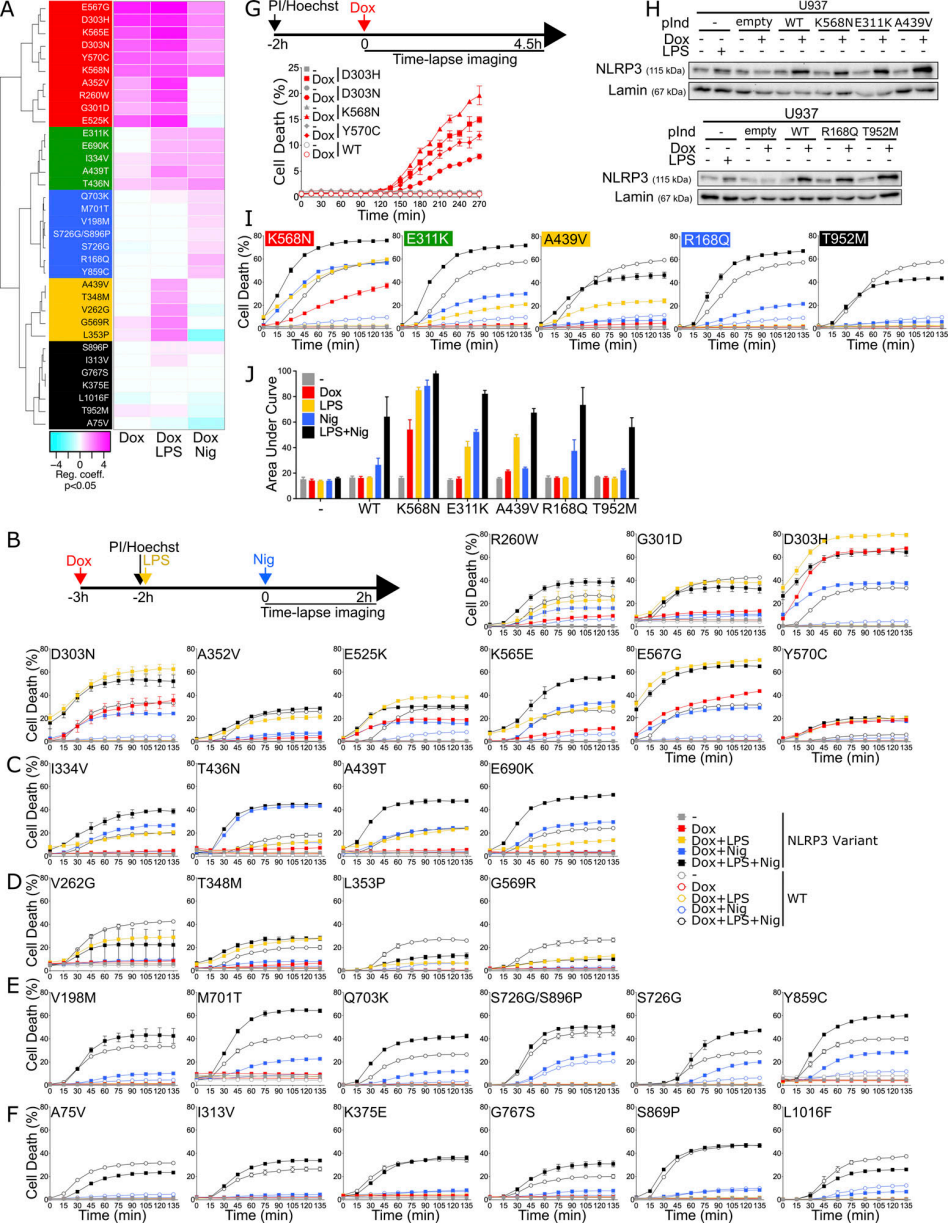
**NLRP3 expression, priming, and/or activation triggers pyroptosis in reconstituted U937 depending on the *NLRP3* variants. (A–F)** *NLRP3*-deficient U937 cells reconstituted with doxycycline-inducible *NLRP3* variants were treated with doxycycline (1 μg/ml, 3 h), LPS (40 ng/ml, 2 h), and nigericin (15 μg/ml) before cell death was monitored by PI incorporation over time quantified by time-lapse high content microscopy. **(A)** Unsupervised clustering of the variants according to RC from the glmm applied on AUC for each variant as compared with the WT. Positive RC denotes increased cell death in the considered variant as compared with the WT, and conversely. RC corresponding to increases (magenta) or decreases (cyan) in cell death are color-coded. Based on the results, variants are classified into five functional groups: constitutive active variants (group#5, red), variants active upon either priming or activation signal (group#4, green), variants active upon priming signal (group#3, yellow), variants active upon activation signal (group#2, blue), and mutants active upon priming and activation signals (no gain-of-function, group#1, black). Results of two to eight independent experiments done in duplicates. **(B)** Group#5. **(C)** Group#4. **(D)** Group#3. **(E)** Group#2. **(F)** Group#1. Means of duplicates and 1 SD are represented. One experiment done in duplicates representative of two to eight independent experiments is shown. Statistical analysis including all independent experiments are represented in Fig. 3. **(G)** *NLRP3*-deficient U937 cells reconstituted with doxycycline-inducible group#5 *NLRP3* variants were treated with doxycycline (1 μg/ml) before cell death was monitored by PI incorporation over time quantified by time-lapse high content microscopy. One experiment done in duplicates representative of two independent experiments is shown. Statistical analysis including all independent experiments is represented in Fig. 2. **(H–J)** U937 cells transduced with vectors encoding indicated doxycycline-inducible *NLRP3* variants or empty vector (empty) and not transduced controls (−) were treated with doxycycline (1 μg/ml, 4 h) or LPS (40 ng/ml, 3 h) and NLRP3 protein levels were analyzed by WB (H). Cells were treated with doxycycline (1 μg/ml, 3 h), LPS (40 ng/ml, 2 h), and/or nigericin (15 μg/ml) before monitoring PI incorporation (I). Means of duplicates and 1 SD are represented. One experiment done in duplicates representative of two to three independent experiments is shown. LDH release was assessed 2 h after nigericin treatment (J). Means of two independent experiments done in duplicates and 1 SD are represented. Source data are available for this figure: SourceData FS2.

The activities of NLRP3 variants were tested under (1) induction of their expression by doxycycline (Dox 3 h), (2) expression induction followed by LPS as a priming signal (Dox 1 h + LPS 2 h), (3) expression induction followed by nigericin as an activation signal (Dox 3 h + Nig), and (4) expression induction followed by both priming and activation signals (Dox 1 h + LPS 2 h + Nig) (Fig. 1 A). We first assessed NLRP3 activity by using pyroptosis as a readout (Fig. 1, Fig. 2, and Fig. S2). We measured propidium iodide (PI) incorporation over time in Hoechst-counterstained cells using high content screening microscopy (Fig. 1 B). The percentage of cell death over time for each variant and after each treatment was compared with that of U937 cells reconstituted with WT *NLRP3* by fitting a linear mixed model (Fig. 2). Using unsupervised clustering, *NLRP3* variants could be classified into five functional groups based on their activation upon induction, priming, and/or activation signals (Fig. S2 A). U937 cells expressing group#5 *NLRP3* variants, such as K568N, underwent cell death upon doxycycline-mediated induction of *NLRP3* expression (Fig. 1 C, Fig. 2, and Fig. S2 B). Group#5 variants corresponded therefore to constitutively active mutants. U937 cells expressing group#4 *NLRP3* variants, like E311K, underwent cell death upon NLRP3 expression and, either priming with LPS or activation with nigericin (Fig. 1 D, Fig. 2, and Fig. S2 C). U937 cells expressing group#3 *NLRP3* variants, like A439V, underwent cell death upon NLRP3 expression and priming with LPS (Fig. 1 E, Fig. 2, and Fig. S2 D). U937 cells expressing group#2 *NLRP3* variants, as R168Q, underwent cell death upon NLRP3 expression and activation with nigericin (Fig. 1 F, Fig. 2, and Fig. S2 E). Finally, U937 cells expressing group#1 *NLRP3* variants, as T952M, underwent cell death upon NLRP3 expression, priming with LPS and activation with nigericin (Fig. 1 G, Fig. 2, and Fig. S2 F). Group#1 variants responded similarly as WT and corresponded to variants with no gain-of-function in reconstituted U937 cells. In our experimental scheme, cell death monitoring commenced when nigericin was added, i.e., 3 h after doxycycline treatment. To investigate early cell death for group#5 *NLRP3* variants, we imaged PI incorporation immediately following doxycycline treatment (Fig. S2 G). Permeabilization of U937 expressing group#5 *NLRP3* variants was detectable starting from 2.5 h following doxycycline treatment, validating our experimental scheme. To test the impact of the co-expression of the WT allele, as typically occurring in patients who are mostly heterozygous, we expressed one variant of each group in U937 cells expressing endogenous NLRP3 (Fig. S2, H and I). The obtained results in U937 were similar, ruling out this potential artifact. Finally, the strong correlation between PI incorporation quantified by microscopy and standard lactate dehydrogenase (LDH) assay confirmed the death of the cells (Fig. S2, I and J).

### Functional screen of *NLRP3* variants using IL-18 and IL-1β secretions as readouts

**Figure 3.**
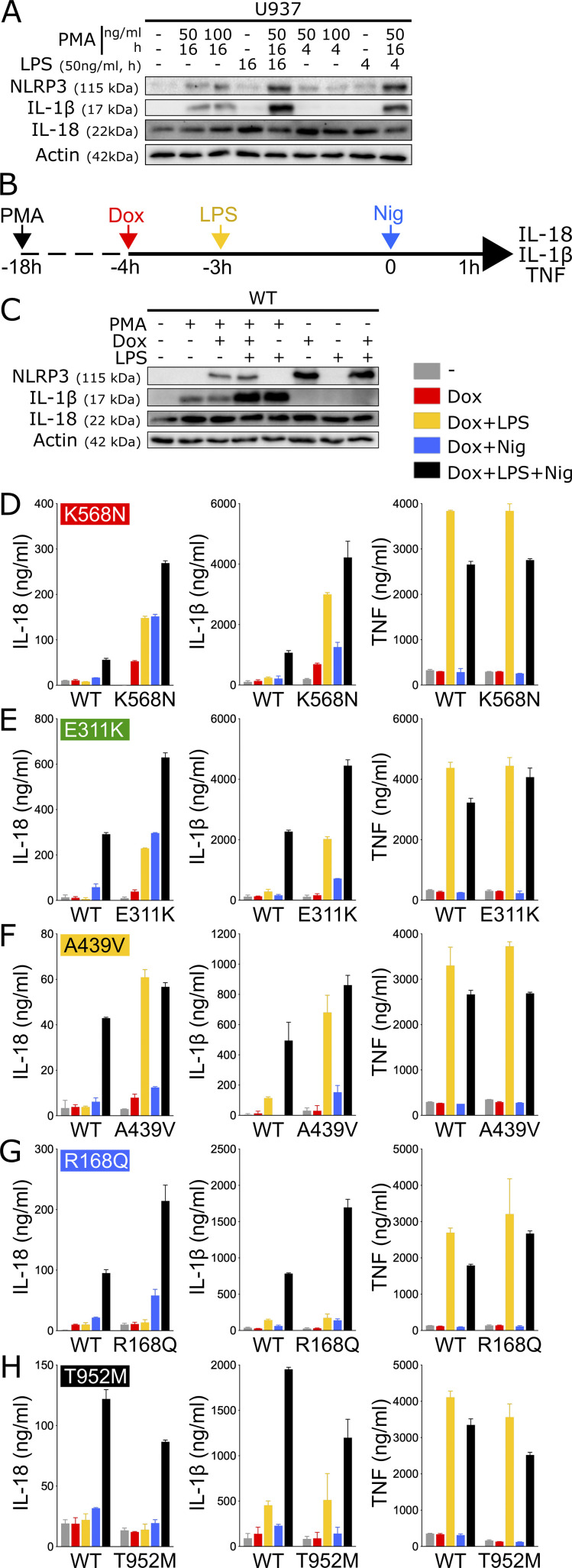
**NLRP3 expression, priming, and/or activation triggers IL-18 and IL-1β secretion in reconstituted U937 depending on the *NLRP3* variants. (A)** U937 cells were differentiated in PMA (50 or 100 ng/ml, 4 or 16 h) and treated with LPS (50 ng/ml, 4 or 16 h). Protein levels of pro-IL-18, pro-IL-1β, and NLRP3 were assessed by WB. Actin is used as loading control. **(B–H)** *NLRP3*-deficient U937 cells reconstituted with doxycycline-inducible *NLRP3* variants were differentiated in PMA (50 ng/ml) and treated with doxycycline (1 μg/ml), LPS (40 ng/ml), and nigericin (15 μg/ml) as indicated. NLRP3, pro-IL-1β, pro-IL-18, and actin levels (as control) were assessed by WB in lysates of *NLRP3*-deficient U937 cells reconstituted with doxycycline-inducible WT *NLRP3* (C). IL-18, IL-1β, and TNF levels (as control) were assessed in cell supernatant by ELISA in K568N (group#5) (D), E311K (group#4) (E), A439V (group#3) (F), R168Q (group#2) (G), and T952M (group#1) (H). Means of duplicates and 1 SD are represented. One experiment done in duplicates representative of 3–18 independent experiments is shown. Results obtained with one variant typical of each functional group are represented. Results obtained with all tested variants and statistical analysis including all independent experiments are represented in Fig. S3. Source data are available for this figure: SourceData F3.

**Figure S3.**
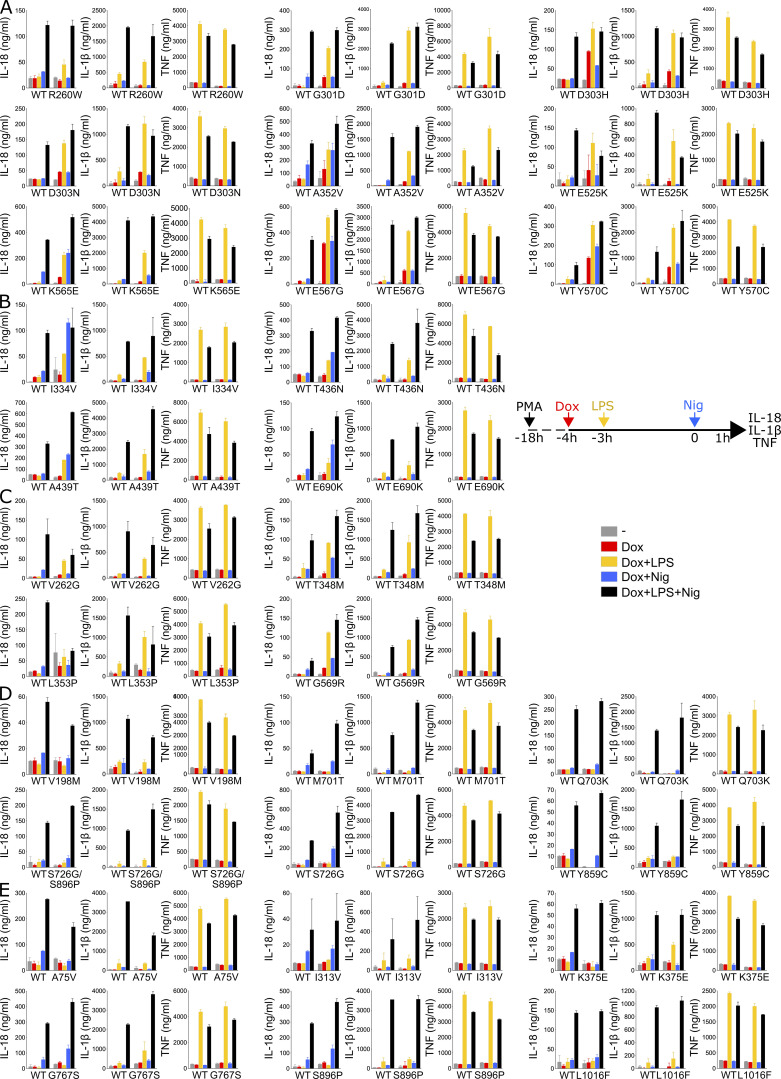
**NLRP3 expression, priming, and/or activation triggers IL-18 and IL-1β secretion in reconstituted U937 depending on the *NLRP3* variants. (A–E)** *NLRP3*-deficient U937 cells reconstituted with doxycycline-inducible *NLRP3* variants were differentiated in PMA (50 ng/ml) and treated with doxycycline (1 μg/ml), LPS (40 ng/ml), and nigericin (15 μg/ml) as indicated. IL-18, IL-1β, and TNF (as control) levels were assessed in cell supernatant by ELISA. Group#5 (A), group#4 (B), group#3 (C), group#2 (D), group#1 (E). Means of duplicates and 1 SD are represented. One experiment done in duplicates representative of 3–18 independent experiments is shown.

**Figure S4.**
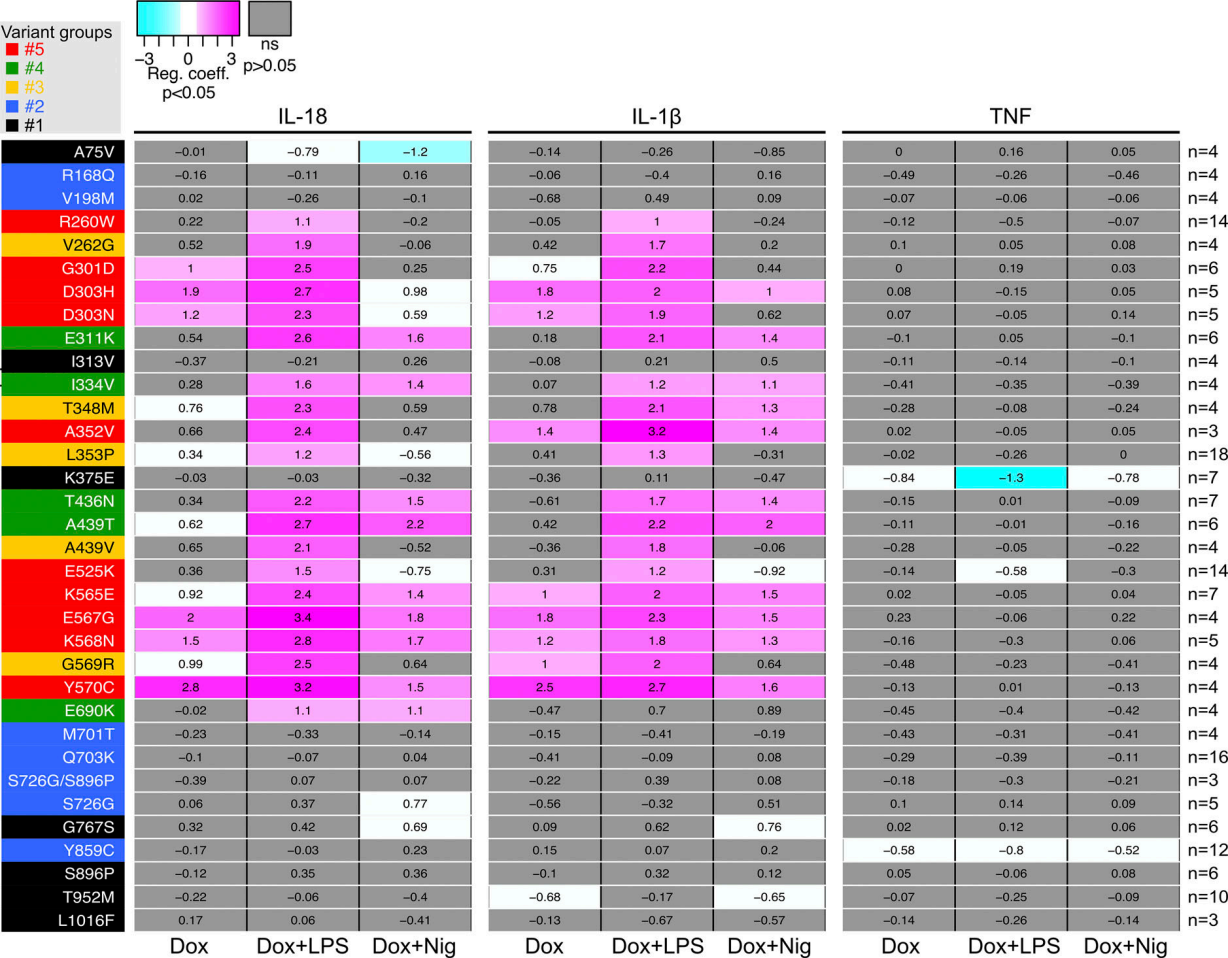
**Statistical analysis of IL-18 and IL-1β secretion in reconstituted U937 upon NLRP3 expression, priming, and/or activation.** RC heatmap from the lmm modeling for each variant as compared with the WT. Positive RC denotes increased cytokine concentrations by the variant as compared to *NLRP3* WT, and conversely. RC corresponding to statistically significant increases (magenta) or decreases (cyan) in cytokine secretions are color-coded (P < 0.05, and RC > +1 or RC < −1, respectively). Not significant variations are color-coded in gray (P > 0.05). Results of 3–18 independent experiments done in duplicates (as indicated for each variant on the right side).

As a complementary approach, we assessed NLRP3 activity by measuring the secretion of inflammasome-dependent cytokines, i.e., IL-18 and IL-1β, following NLRP3 induction, priming, and activation. LPS priming did not induce pro-IL-1β expression in U937 monocytes, while pro-IL-1β was induced in PMA-differentiated U937 cells (Fig. 3 A). Pro-IL-18 was expressed in basal conditions in U937 as expected and was not increased upon PMA differentiation and LPS priming. We therefore measured IL-18 and IL-1β secretion in U937 macrophages differentiated with PMA 50 ng/ml for 16 h, followed by treatments with doxycycline, LPS, and/or nigericin (Fig. 3, B–H and Fig. S3, A–E). TNF was measured as a control for NLRP3-independent cytokine secretion. IL-18, IL-1β, and TNF secretions for each variant following each treatment were compared with those of U937 cells reconstituted with WT *NLRP3* using linear mixed-effects models (Fig. S4). Notably, PMA differentiation was associated with low expression of pro-IL-1β in reconstituted U937 *NLRP3* KO cells in the absence of LPS priming and lower induction of *NLRP3* following doxycycline treatment as compared with undifferentiated cells (Fig. 3 C).

For all variants, IL-1β secretion was higher in magnitude than IL-18 secretion and constituted a more consistent readout. For most *NLRP3* variants, IL-18 and IL-1β secretion matched the induction of pyroptosis in response to NLRP3 expression, priming, and activation signals in reconstituted U937 cells. Cells expressing group#5 *NLRP3* variants, such as K568N, secreted IL-18 and IL-1β in response to doxycycline (Fig. 3 D and Fig. S3 A). U937 cells expressing group#4 *NLRP3* variants, like E311K, secreted IL-18 and IL-1β in response to either LPS priming or activation with nigericin (Fig. 3 E and Fig. S3 B). U937 cells expressing group#3 *NLRP3* variants, like A439V, secreted IL-18 and IL-1β in response to LPS priming (Fig. 3 F and Fig. S3 C). U937 cells expressing group#2 *NLRP3* variants, for example, R168Q, secreted IL-18 in response to the nigericin activation signal (Fig. 3 G and Fig. S3 D). Finally, U937 cells expressing group#1 *NLRP3* variants, for example, T952M, secreted IL-18 and IL-1β only in response to priming and activation signals (Fig. 3 H and Fig. S3 E).

In LPS-primed conditions, the statistical test robustly detected increased IL-18/1β secretion for all variants of groups#5, #4, and #3 compared with U937 cells expressing WT *NLRP3* (Fig. S4). Nevertheless, in the absence of LPS priming, several discrepancies could be noted between IL-18/1β secretion and pyroptosis. U937 cells expressing group#5 *NLRP3* variants R260W and E525K did not robustly secrete IL-18/1β in response to doxycycline, while they underwent pyroptosis. Similarly, U937 cells expressing group#5 *NLRP3* variants R260W, G301D, D303N, and E525K as well as U937 cells expressing all group#2 *NLRP3* variants did not robustly secrete IL-18/1β in response to doxycycline and nigericin, while they underwent pyroptosis. We might speculate that lower NLRP3 expression in PMA-differentiated U937 cells in response to doxycycline and low expression of pro-IL-1β in these unprimed conditions may explain the lower sensitivity of the IL-18/1β secretion readout. On the opposite, U937 cells expressing group#3 *NLRP3* variant T348M secreted IL-1β in response to doxycycline and nigericin, indicating that this variant may exhibit partial activity that may lead to IL-18 and IL-1β secretion without cell death in this condition. As a control, U937 cells reconstituted with *NLRP3* variants secreted a similar amount of TNF as U937 cells reconstituted with WT *NLRP3* in all conditions (Fig. S4).

### Sensitivity of *NLRP3* variants to NLRP3 chemical inhibitors

**Figure S5.**
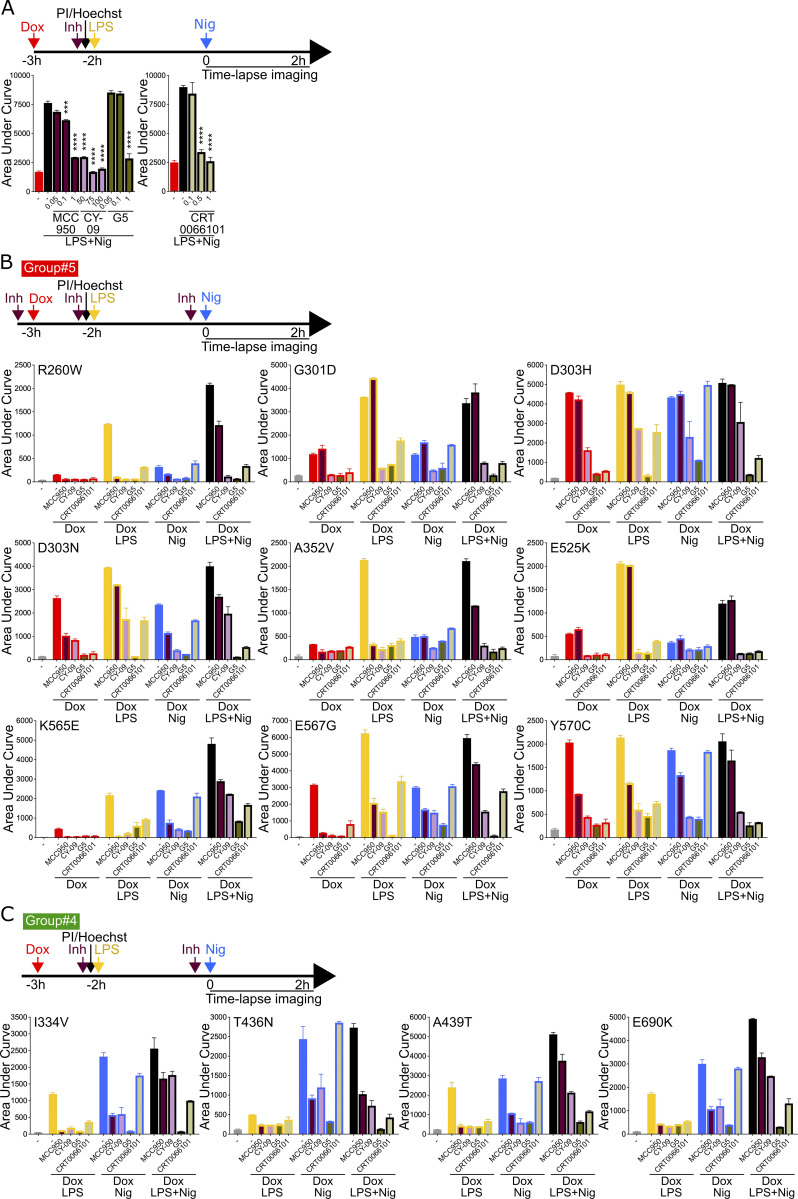
**Sensitivity of *NLRP3* variants to NLRP3 inhibitors (groups#5 and 4). (A)** Dose-dependent inhibition of pyroptosis by NLRP3 inhibitors (Inh). *NLRP3*-deficient U937 cells reconstituted with doxycycline-inducible WT *NLRP3* and treated with doxycycline (1 μg/ml, 3 h) were treated with indicated doses (μM) of MCC950, CY-09, G5, and CRT0066101 or vehicle (2h15), LPS (40 ng/ml, 2 h), and nigericin (15 μg/ml) before cell death was monitored by PI incorporation over time quantified by time-lapse high content microscopy. Means of AUC of duplicates and 1 SD are represented. Two-way ANOVA multiple comparisons of each variant with WT control with corresponding treatment, ***, P < 0.001; ****, P < 0.0001. **(B and C)** *NLRP3*-deficient U937 cells reconstituted with doxycycline-inducible *NLRP3* variants were treated with doxycycline (1 μg/ml, 3 h), LPS (40 ng/ml, 2 h), and/or nigericin (15 μg/ml) in the presence of MCC950 (1 μM), CY-09 (50 μM), G5 (1 μM), and CRT006101 (0.5 μM) NLRP3 inhibitors or DMSO vehicle (added 20 min before the last treatment), and cell death was monitored by PI incorporation over time quantified by time-lapse high content microscopy for 2 h. Group#5 (B), group#4 (C). Means of AUC of duplicates and 1 SD are represented. One experiment done in duplicates representative of two independent experiments is shown. Statistical analysis including all independent experiments are represented in Fig. 4 H.

**Figure 4.**
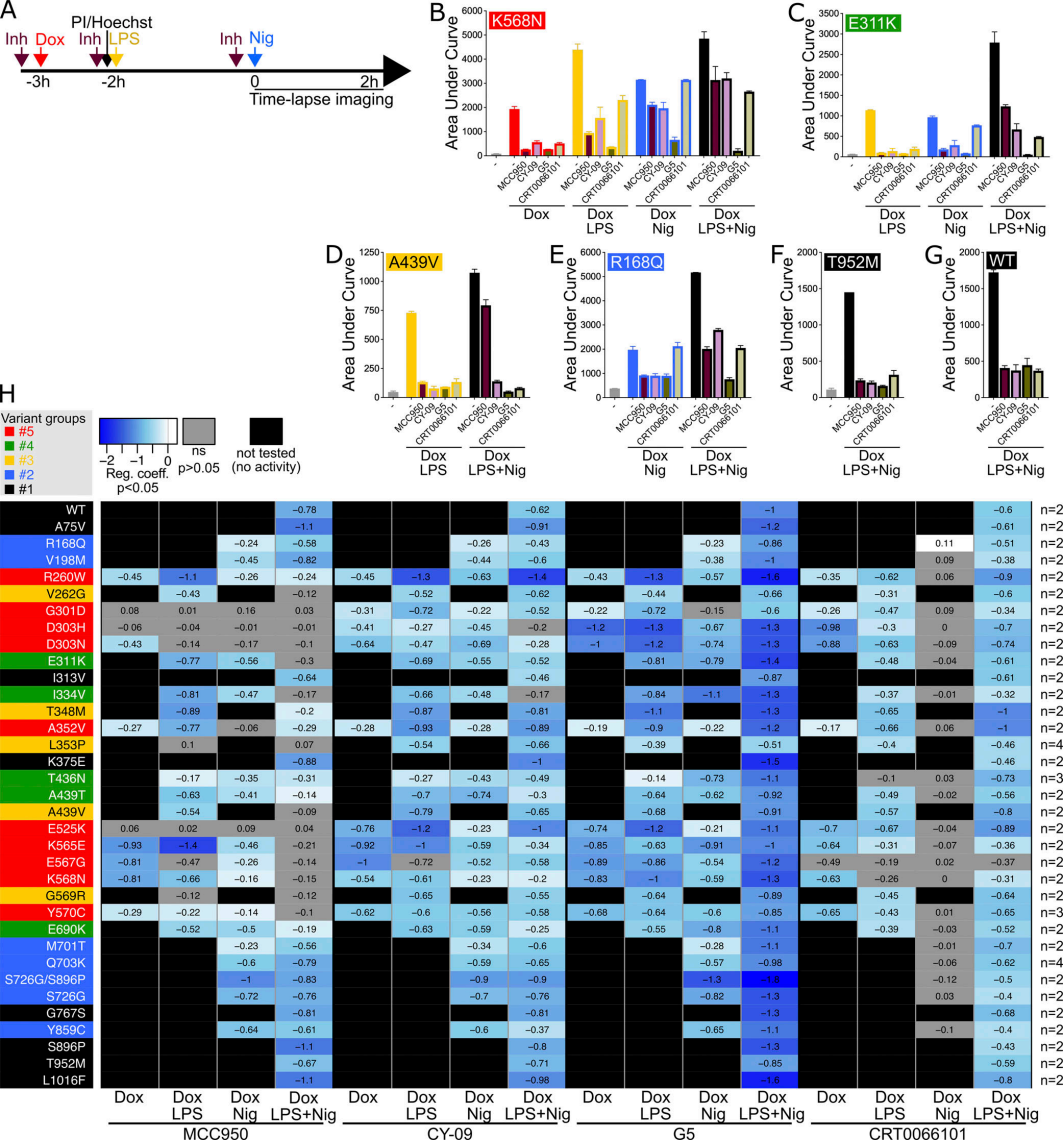
**Cell death of U937 expressing *NLRP3* variants in response to NLRP3 expression, priming, and/or activation in the presence of NLRP3 inhibitors. (A–G)** *NLRP3*-deficient U937 cells reconstituted with doxycycline-inducible *NLRP3* variants were treated with doxycycline (1 μg/ml, 3 h), LPS (40 ng/ml, 2 h), and nigericin (15 μg/ml) in the presence of MCC950 (1 μM), CY-09 (50 μM), G5 (1 μM), and CRT006101 (0.5 μM) NLRP3 inhibitors (Inh), or DMSO vehicle (added 20 min before the last treatment) and cell death was monitored by PI incorporation over time quantified by time-lapse high content microscopy for 2 h (A). **(B)** K568N (group#5). **(C)** E311K (group#4). **(D)** A439V (group#3). **(E)** R168Q (group#2). **(F)** T952M (group#1). **(G)** WT as control. Means of AUC of duplicates and 1 SD are represented. One experiment done in duplicates representative of two independent experiments is shown. Results obtained with one variant typical of each functional group are represented (B–F). Results obtained with all tested variants are presented in Fig. S4, B–F. **(H)** Statistical analysis of the inhibition of each variant in each relevant condition by MCC950, CY-09, G5, and CRT0066101 compounds. Inhibition heatmap from the linear mixed model of PI incorporation AUC for each *NLRP3* variant with a specific treatment for each inhibitor, as compared with vehicle only. Inhibition is expressed as RC corresponding to Log10(1−%inhibition/100). The negative coefficient denoted inhibition of cell death, and the null coefficient corresponded to no inhibition. For significant values (P < 0.05), RC between −2 (99% inhibition) and 0.1 (20% inhibition) are color-coded with decreasing blue intensities. In gray, not significant values (P > 0.05) correspond to conditions (variant * treatment) in which the inhibitors do not decrease PI incorporation. In black, the variant * treatment combinations do not trigger cell death and therefore inhibitors were not tested. Results of two independent experiments done in duplicates.

**Figure S6.**
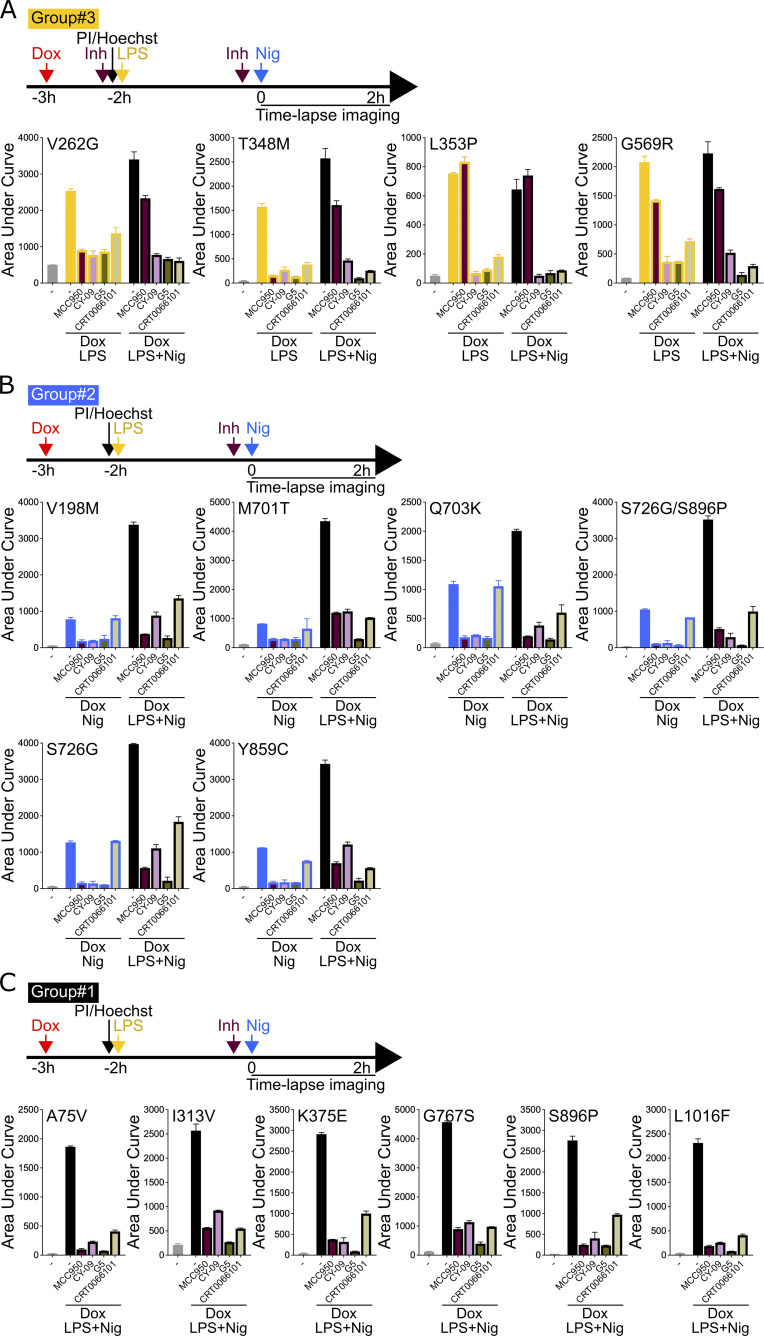
**Sensitivity of *NLRP3* variants to NLRP3 inhibitors (groups#3–1). (A–C)** *NLRP3*-deficient U937 cells reconstituted with doxycycline-inducible *NLRP3* variants were treated with doxycycline (1 μg/ml, 3 h), LPS (40 ng/ml, 2 h), and/or nigericin (15 μg/ml) in the presence of MCC950 (1 μM), CY-09 (50 μM), G5 (1 μM), and CRT006101 (0.5 μM) NLRP3 inhibitors (Inh) or DMSO vehicle (added 20 min before the last treatment), and cell death was monitored by PI incorporation over time quantified by time-lapse high content microscopy for 2 h. Group#3 (A), group#2 (B), and group#1 (C). Means of AUC of duplicates and 1 SD are represented. One experiment done in duplicates representative of two independent experiments is shown. Statistical analysis including all independent experiments are represented in Fig. 4 H.

We next investigated the sensitivity of the *NLRP3* variants to inflammasome inhibitors. We tested direct NLRP3 inhibitors MCC950 and CY-09 (Coll et al., 2015; Jiang et al., 2017). MCC950 interacts with NLRP3 into a cleft in between the NACHT, (NAIP, CIITA, HET-E, and TEP1) and the LRR (leucin-rich repeats) domains in proximity to the ATP-binding site and locks NLRP3 in an inactive conformation (Hochheiser et al., 2022; Brinkschulte et al., 2022; Tapia-Abellán et al., 2019; Dekker et al., 2021). CY-09 binding to NLRP3 depends on the integrity of the NLRP3 ATP-binding site and its inhibitory mechanism remains debated (Jiang et al., 2017; Brinkschulte et al., 2022). We also tested two indirect inhibitors: the deubiquitinase inhibitor G5, which impairs NLRP3 deubiquitination by BRCC3 (Py et al., 2013), and the protein kinase D inhibitor CRT0066101, which impairs NLRP3 phosphorylation at the Golgi during the activation process (Zhang et al., 2017). We determined the optimal dose of each inhibitor using *NLRP3*-deficient U937 reconstituted with WT *NLRP3* treated with doxycycline, LPS, and nigericin to reach similar 80% inhibition for all tested inhibitors (Fig. S5 A). We then measured cell death following Dox (group#5), Dox+LPS (groups#3–5), Dox+Nig (groups#2, 4, 5), or Dox+LPS+Nig (groups#1–5) in the presence of these inhibitors added 20 min before the last treatment for each condition (Fig. 4, B–G; Fig. S5, B and C; and Fig. S6). For each variant and each treatment, we compared cell death in the presence of each of the inhibitors to vehicle only by fitting a linear mixed-effects model to area under the curve (AUC) value databases (Fig. 4 H). None of the group#1 variants showed reduced sensitivity to any of the inhibitors, confirming the absence of gain-of-function of these variants.

**Figure S7.**
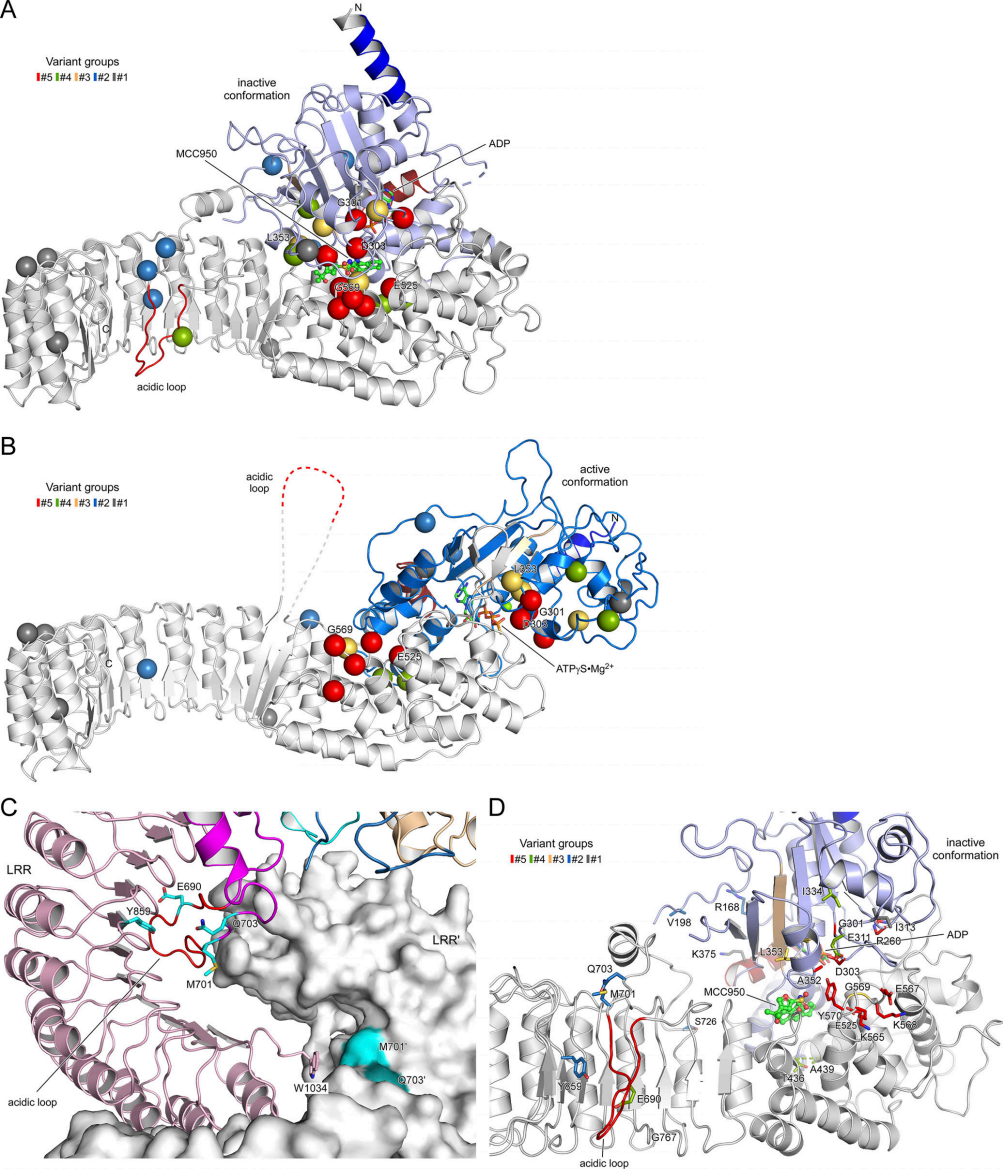
**Details of the disease-causing mutant sites in NLRP3 and MCC950 inhibition. (A)** Localization of gain-of-function mutations in the MCC950-inhibited state of human NLRP3 (7PZC), color-coded according to variant groups. The five mutant positions that are not responsive to MCC950 treatment, G301D, E303H, L353P, E525K, and G569R, are labeled. **(B)** Localization of gain-of-function mutations in the adenosine triphosphate-bound active state of human NLRP3 (8EJ4). **(C)** The three gain-of-function mutations in the acidic loop of NLRP3, E690, M701, and Q703, are in the dimer interface of the interlaced LRRs, while Y859 in the concave surface of the LRR interacts with the acidic loop. **(D)** MCC950 is in the center of the NACHT and LRR domains in proximity to many disease-causing mutations.

Most gain-of-function mutants are sensitive to MCC950, including all the mutants of group#2. Nevertheless, consistent with previous studies, several *NLRP3* mutants were resistant (Vande Walle et al., 2019; Coll et al., 2015; Dekker et al., 2021). G301D, D303H, and E525K (group#5) as well as L353P and G569R (group#3) were resistant to MCC950 independently of the signals (priming or activation) provided. These mutations may affect NLRP3’s ability to bind to MCC950 or impair the ability of MCC950 to lock NLRP3 in a closed conformation (Fig. S7, A and D). I334V, A439T, and E690K (group#4), and V262G, A439V, and T348M (group#3) were sensitive to MCC950 when added before the priming signal, but were resistant to MCC950 when added on primed U937 before the activation signal. In this last condition, these mutants may switch to an active conformation upon priming before the addition of MCC950. Similarly, K565E, E567G, K568N, and Y570C (group#5) were resistant to MCC950, when added before the activation signal on primed cells. Noteworthy, K568N and Y570C were also partly resistant to MCC950 when added before the activation signal on non-primed cells.

E311K, I334V, A439T, and E690K (group#4) were inhibited by MCC950 when added before priming or activation signal on non-primed cells, while T436N (group#4) was resistant to MCC950 when added before the priming signal, but sensitive to MCC950 when added before the activation signal. This suggested that while both priming and activation signals resulted in active T436N form, the structural switches underlying this activation process may differ. R260W (group#5) was inhibited by MCC950 when added before the LPS priming signal or before the activation signal on primed cells. Although MCC950 was much less potent in this last condition, this may indicate that the LPS-induced structural switch in these mutants was reversible and that MCC950 may lock back NLRP3 in a closed conformation. D303N (group#5) was sensitive to MCC950 when added before its protein induction, but resistant if added later before the priming or the activation signal, suggesting that upon induction, D303N may transiently adopt a closed conformation stabilized by MCC950, but quickly switch to an irreversible active conformation which may be stabilized by its oligomerization. In contrast to MCC950, no *NLRP3* variants showed strong resistance to the direct inhibitor CY-09. E525K, K568N (group#5), and R168Q (group#2) showed partial resistance to CY-09 when added before the activation signal to non-primed cells. D303H (group#5) and T436N (group#4) showed partial resistance when CY-09 was added before the priming signal. A352V (group#5) showed partial resistance when CY-09 was added upon induction or before the activation signal.

Concerning the indirect inhibitors, no *NLRP3* variants showed strong resistance to the deubiquitinase inhibitor G5. E525K (group#5) and R168Q (group#2) showed partial resistance when G5 was added before the activation signal to non-primed cells. A352V (group#5) was partially resistant when G5 was added before the induction or the activation signal. T436N (group#4) was partially resistant when G5 was added before the priming signal. CRT0066101 was inefficient in inhibiting any mutant of groups#5, #4, or #2 when added before the activation signal in non-primed cells. In addition, E567G (group#5) and T436N (group#4) were resistant to CRT0066101 when added before priming, and A352V (group#5) was partially resistant to CRT0066101 when added upon induction. Noteworthy, observed resistance to all inhibitors of A352V (if added before induction or activation of unprimed cells), T436N (if added before priming), and E525K (if added before activation of unprimed cells) may be artifactual as these mutants showed the weakest gain-of-function of their groups in these conditions (Fig. 2).

### Activity of *NLRP3* variants in patient primary monocytes

**Figure 5.**
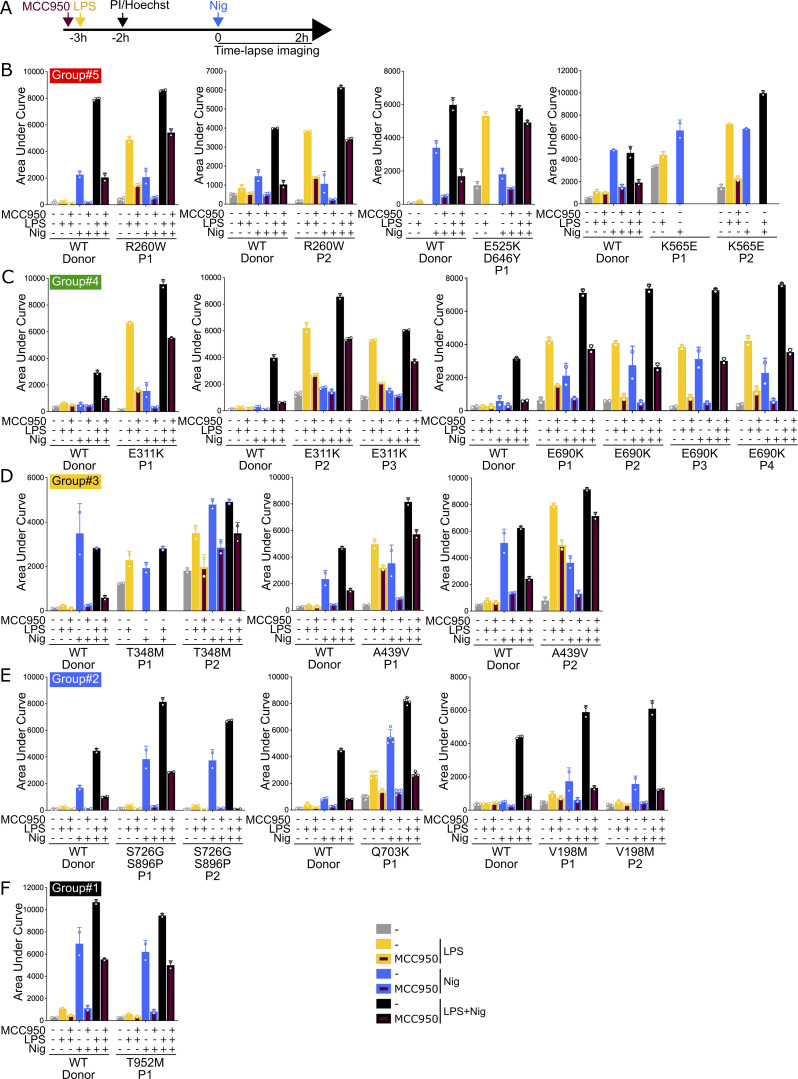
**Patient monocytes respond differently to NLRP3 priming and/or activation, and NLRP3 inhibitor MCC950 depending on *NLRP3* variants. (A)** Monocytes of 22 autoinflammatory patients and healthy donors (WT) were treated with LPS (40 ng/ml, 3 h) and nigericin (5 μg/ml) in the presence of MCC950 (1 μM, added 15 min before LPS), and cell death was monitored by PI incorporation over time quantified by time-lapse high content microscopy for 2 h. **(B)** R260W, E525K+D646Y, and K565E (group#5). **(C)** E311K and E690K (group#4). **(D)** T348M and A439V (group#3). **(E)** S726G+S896P, Q703K, and V198M (group#2). **(F)** T952M (group#1). Means of AUC of two to six replicates and 1 SD are represented. One experiment done in two to six replicates is shown. Data represented in the same graph correspond to one same experiment on relatives. Due to limited amount of blood from patients E525K/D646Y P1, K565E P1 and P2, and T348M P1, some conditions could not be tested.

In addition, we developed a functional assay on primary monocytes adapted for small-volume pediatric blood samples as it requires <0.2 × 10^6^ monocytes. We assessed pyroptosis of primary monocytes from 22 patients bearing *NLRP3* variants in response to LPS and/or nigericin, and its sensitivity to MCC950 inhibition (Fig. 5). Priming and activation signals have both been independently described to be dispensable for NLRP3 activation in human monocytes (Gaidt et al., 2016; Gritsenko et al., 2020). In our experimental settings (dose, kinetics), LPS priming alone did not cause pyroptosis in monocytes from healthy donors. In the absence of priming, nigericin triggered pyroptosis in a MCC950-sensitive manner although with slower kinetics than in the presence of priming. In this context, additional priming signals from death of neighboring cells upon transportation or monocytes’ purification procedures could not be excluded.

NLRP3 induction and priming could not be discriminated in this experimental setting, and monocytes from patients bearing *NLRP3* variants of group#5 (R260W, E525K+D646Y, and K565E), group#4 (E311K and E690K), and group#3 (T348M and A439V) showed increased pyroptosis in response to LPS as compared with their respective control monocytes from healthy donors (Fig. 5, B–D). Monocytes from patients bearing *NLRP3* variants of group#4 (E311K and E690K) and group#2 (S726G+S896P, Q703K, and V198M) showed increased pyroptosis in response to nigericin (Fig. 5, C and E). Monocytes from a patient bearing an *NLRP3* variant of group#1 (T952M) showed no gain-of-function relative to their controls (Fig. 5 F). MCC950 inhibited pyroptosis of monocytes from all patients, except those from the patient bearing E525K (Fig. 5, B–F), consistent with inhibition assays on reconstituted U937 cells (Fig. 4 H).

## Discussion

The clinical manifestations of NLRP3-AID are diverse and most of them are poorly specific. Diagnosis relies largely on the identification of *NLRP3* variants by genetic analysis, but only 11% of known variants have been fully characterized as benign or pathogenic (Infevers 20/12/2023), while the functional link to the disease remains to be established for all the others (Van Gijn et al., 2018). In addition, gain-of-functions of *NLRP3* variants have been assessed by monitoring NLRP3 activity in response to priming signals, which does not distinguish between constitutive and priming-induced activity, and NLRP3 activity in response to activation signals in the absence of priming has not been considered (Rieber et al., 2015). By reconstitution of *NLRP3*-deficient U937 monocytes, we functionally characterized 34 *NLRP3* variants, including the most frequent in France, on an identical genetic background. For 11 variants, results were confirmed on primary monocytes from 22 patients. We highlighted the functional diversity of *NLRP3* variants, which we classified into five groups. Identification of *NLRP3* variants with no gain-of-function (group#1) will exclude NLRP3-AID diagnosis and guide decision toward broader genetic analysis for patients positive for these variants after gene panel approaches. Genotype–phenotype correlation is difficult to assess. Indeed, severities vary between patients bearing identical mutations, and in the era of anti-IL-1 therapy, delayed access to treatment rather than the genotype determines symptom severity. Nevertheless, the low genotype overlap between somatic and germline mutations suggests that somatic mutations, which may be responsible for 0.5–19% of NLRP3-AID cases, would be highly detrimental and incompatible with life if germline. Interestingly, the functional diversity identified in our study strongly correlates with somatic vs. germline mutations. All somatic mutations included in our study (*n* = 5) correspond to constitutively active mutants (group#5, *n* = 4) or mutants activated by priming or activation signal (group#4, *n* = 1). 40% of group#5 mutants are associated with mosaicism (4/10), 20% of group#4 (1/5), but none of groups#3 and #2 (0/11).

Comparing the sensitivity of *NLRP3* mutants to two direct and two indirect inhibitors revealed that the direct inhibitor CY-09 was efficient on more variants than MCC950, and that the deubiquitinase inhibitor G5 was efficient on all mutants. This suggests that refining the modes of action of these compounds may be of particular interest for developing potent inhibitors with therapeutic potential against most NLRP3-AID mutants. In this line, inhibitors of the BRCC3 deubiquitinase that directly target NLRP3 show promising results in several mouse inflammation models (Ren et al., 2021).

Functional assays on primary patients’ monocytes face limitations due to ethical issues related to blood drawn from pediatric patients and technical challenges related to the low number of monocytes purified from pediatric samples, the cell stress caused by transportation, and the need for rapid delivery and processing of clinical samples. Reconstituting NLRP3-deficient U937 lines with doxycycline-inducible *NLRP3* variants enabled the comparison of *NLRP3* variants on an identical genetic background and distinguished NLRP3 constitutive activity from priming-induced and/or activation-induced activity. Notably, expression levels were similar for most of the *NLRP3* variants, excluding any technical artifacts caused by overexpression of some mutants. In contrast, the two mutants with the lowest expressions, E567G and D303N, were found constitutively active, suggesting that their lower expressions likely resulted from their cytotoxic activity. Time-lapse assessment of pyroptosis at the single-cell level proved to be a very sensitive and robust readout. Compared with cytokine secretion assays, it required lower numbers of cells and could be applied to monocytes purified from pediatric autoinflammatory patients. IL-18 secretion was poorly reproducible due to its low magnitude and IL-1β was produced only after PMA differentiation in U937 cell lines. Because PMA differentiation lowered NLRP3 expression levels and partially provided the priming signal, cytokine secretions appeared to be less robust readouts. Nevertheless, they confirmed most of the results of pyroptosis assays. Differently from primary monocytes and THP1 or BlaER1 lines, U937 cells did not undergo pyroptosis upon nigericin treatment. This facilitated the detection of gain-of-functions that could bypass the requirement for priming, which were later confirmed in primary monocytes.

In our study, we describe five functional groups of *NLRP3* variants, classified according to their cellular response to induction, priming, and/or activation signals. This led to the definition of a new group of variants, group#2, responding to the activation signal in the absence of priming. The clinical significance of these variants has been long debated due to their low penetrance and high allele frequency in healthy populations. Our data are in agreement with previous studies reporting their unresponsiveness to priming signals, but highlight their response to the activation signal in the absence of priming reconciliating results of in vitro assays with the higher prevalence of these variants in autoinflammatory patients (Rieber et al., 2015; Theodoropoulou et al., 2020). Altogether, the data suggested that the group#2 variants act as susceptibility alleles to inflammation through a different mechanism than other NLRP3-AID mutations. Consistently, group#2 variants are often associated with symptoms atypical for NLRP3-AID and lower response to anti-IL-1 therapy (Fayand et al., 2023; Kuemmerle-Deschner et al., 2017). These findings should be considered for atypical patient diagnosis toward NLRP3-AID and choices of therapy. Additional studies combining cohort analysis and mechanistic approaches would be required to investigate the link between the group and the atypical symptoms or response to treatment. Gain-of-function of most group#2 variants in response to Dox+Nig was of low magnitude and could be observed using pyroptosis but not cytokine secretion readouts (R168Q, V198M, M701T, Q703K, S726G, Y859C), in line with the incomplete penetrance of these mutations. Noteworthy, some group#1 variants (K375E, G767S, S896P), not considered as gain-of-function based on our analysis, showed trends toward increased pyroptosis in response to Dox+Nig, albeit below the significance threshold. The sensitivity of our assays to statistically detect gain-of-function mutations increases with the number of independent experiments included for one given variant. Therefore, deeper analysis with more replicates might conclude to slight gain-of-function of these variants. Identification of constitutively active mutants (group#5) by treatment of reconstituted U937 with doxycycline indicated that other key proteins of the pathway (apoptosis-associated speck-like protein containing a CARD, caspase-1, GSDMD) pre-existed in functional forms in U937, and that priming and activation signals acted on NLRP3 itself.

**Figure 6.**
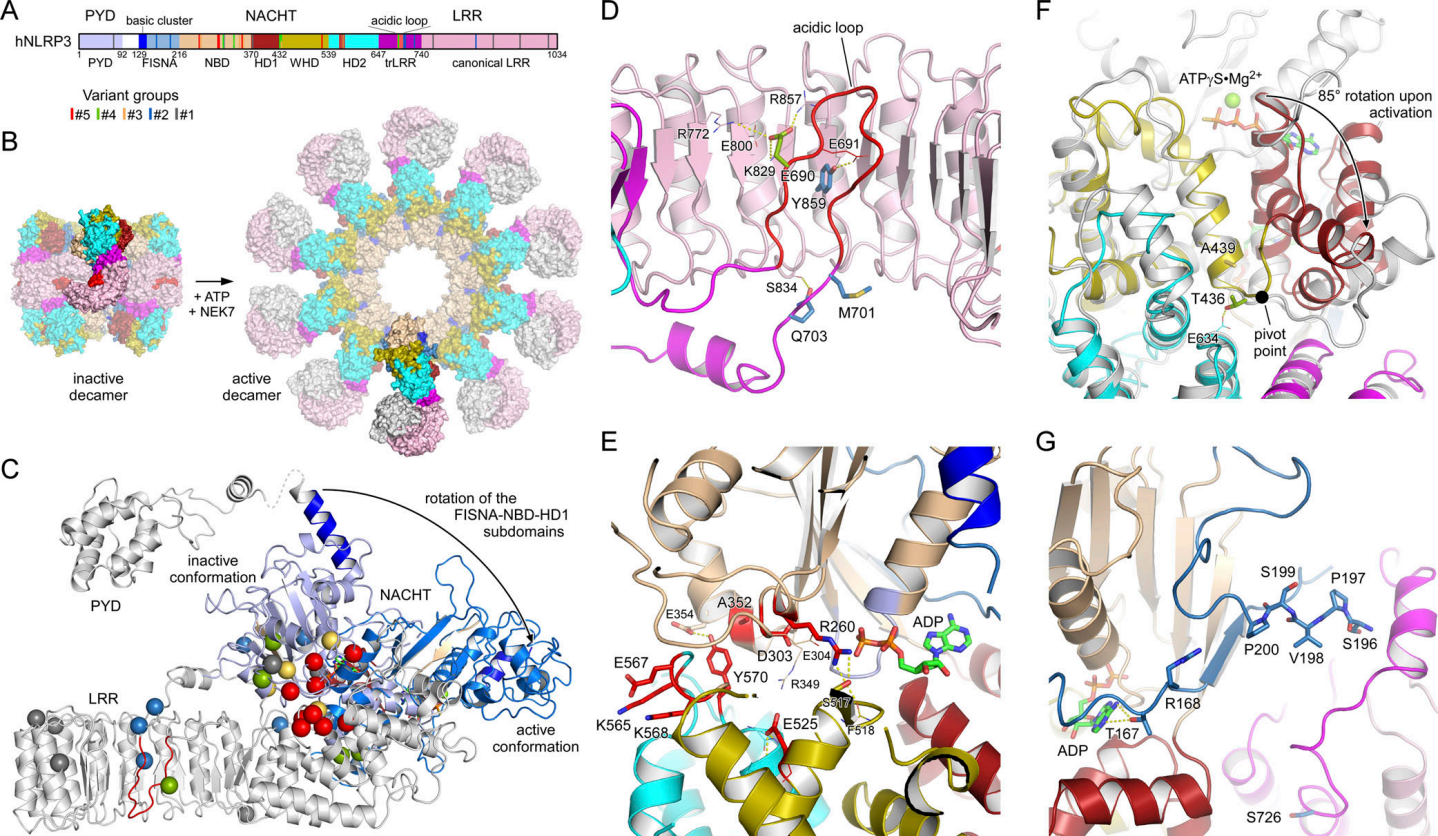
**Localization of disease mutant sites in the structure of human NLRP3. (A)** Bar diagram showing the domain architecture of NLRP3 with the mutant sites of the five variant groups indicated. **(B)** Display of the inactive spherical NLRP3-ADP decamer (7PZC) (Hochheiser et al., 2022) and the active radial NLRP3-ATP decamer bound to NEK7 (in gray) (8EJ4) (Xiao et al., 2023). **(C)** The conformational transition from the inactive to the active state in NLRP3 involves an 85° rotation of the FISNA-NBD-HD1 subdomains in the NACHT relative to the remainder. The missense mutations of the five variant groups are highlighted as spheres in the inactive conformation. **(D)** Close-up of residues E690, M701, and Q703 in the loop section of the transition LRR and Y859 in the concave side of the LRR. **(E)** Localization of the constitutively active group#5 mutant sites in the NACHT domain of NLRP3. **(F)** Residues T436 and A439 are at the pivot point of the FISNA-NBD-HD1 rotation. The active state (8EJ4) is shown in gray relative to the colored inactive state (7PZC). **(G)** Variant group#2 residues R168 and V198 in the FISNA domain and S726 in the trLRR are located in variable regions of NLRP3.

Given the recent advances in NLRP3 structure determination, the identification of gain-of-function mutations provides insights into the key regulatory mechanisms of inflammasome assembly. A bar diagram depicts the localization of mutant sites according to the domain architecture of NLRP3 (Fig. 6 A). Based on our current knowledge, the transition from the inactive spherical decamer to the active radial decamer requires the release of the LRR domains by binding to NEK7 (Fig. 6 B). This conformational transition goes along with an 85° rotation of the nucleotide-binding domain (NBD) and helical domain 1 (HD1) subdomains relative to the winged helix domain (WHD) and HD2 subdomains within the NACHT, which is the central mechanism of the activation of signal transduction ATPases with numerous domains and accompanied by a nucleotide exchange from ADP to ATP (Fig. 6 C and Fig. S7, A and B) (Hochheiser et al., 2022; Xiao et al., 2023). Some disease mutations may destabilize the inactive spherical “cage” structure with the pyrin domain effector domains buried inside, which relies mostly on interactions between the LRR domains (Andreeva et al., 2021; Hochheiser et al., 2022; Ohto et al., 2022). In human NLRP3, a loop section of 42 residues mediates the interlaced LRR dimer assembly with the acidic loop binding on one side into the concave side of its LRR structure and on the other side to the tip of the cognate LRR (Fig. 6 D and Fig. S7 C). Y859 maps to the concave side of the LRR directly interacting with the acidic loop (687–700) comprising E690, suggesting that these two NLRP3-AID mutation sites may destabilize the inactive spherical structure (Hochheiser et al., 2022). Residues M701 and Q703 from group#2 similarly reach out into the concave side of the canonical LRR, and any mutation here might influence the intertwined LRR dimer assembly.

The group#5 variants of constitutively active mutants are all located in the NACHT domain in proximity to the nucleotide-binding site. D303 is in the Walker B motif with the typical aaaaDE motif degenerated to aaaaDGxD_303_E in NLRs (where “a” denotes an aliphatic residue) (Brinkschulte et al., 2022). The acidic residues catalyze the intrinsic ATP hydrolysis mechanism that is required for the relapse from an active to an inactive state, and it is well-perceived that any mutation in this motif enriches the active state of NLRP3. A352 follows the sensor 1 motif (R349) on β-strand 4, interacting with D303 on β-strand 3 from the back (Fig. 6 E). Its mutation to a larger residue could also disrupt the inactivation mechanism. The R260 residue at the tip of β-strand 2 is buried in the structure of the inactive NACHT domain (PDB: 7PZC) but on the surface close to the γ-phosphate group in the active NACHT conformation (PDB: 8EJ4). Its mutation to a larger Trp-residue might prevent the formation of the tightly closed inactive state. The acidic E525 in WHD forms a salt bridge with the sensor 1 R349 following H-bonds with HD2. A charge reversal to Lys will dismiss these interactions. Finally, residues K565, E567, and K568 are on the accessible surface of the HD2 domain, both in the inactive and active state. Their disease-related mutations point to an important surface patch on this side. The succeeding residue Y570 is again inside and interacts with the NBD, yet its mutation to Cys could indirectly influence the three preceding residues. This similarly holds for the G569R mutation of group#3, which might also affect the integrity of the K_565_xEKGY stretch.

The group#5 G301D mutant is proximate to D303 and part of the degenerated Walker B motif. The G301E mutation has been shown to have an eightfold reduced hydrolysis activity, enriching thus the active state. Residues T436 and A439 from groups #4 and #3 are located in the WHD near the pivot point, switching between the inactive and active conformations (Fig. 6 F) (Hochheiser et al., 2022; Xiao et al., 2023). The new group#2 disease variants R168 and V198 are both located in the fish-specific NACHT associated (FISNA) domain. R168 is next to T167 that interacts with the adenine base of the bound nucleotide (Fig. 6 G). V198M may stabilize the FISNA domain in the active conformation, as V198 is located in the activation loop comprising S_196_PVSP, whose priming-associated phosphorylation is likely to destabilize the inactive cage complex (Hochheiser et al., 2022; Xiao et al., 2023).

MCC950 binds NLRP3 in a cleft spanned by subdomains NBD, HD1, WHD, HD2, and transition LRR (trLRR), stabilizing the inactive conformation of the spherical decamer (Fig. S7 D) (Hochheiser et al., 2022). Our results on the inhibition of cell death upon NLRP3 WT or NLRP3-AID variant expressions confirmed the sensitivity to MCC950 of V198M, E311K, T348M, the partial sensitivity of A352V, A439V, D303N, and the full resistance of L353P (Coll et al., 2015; Tapia-Abellán et al., 2019; Vande Walle et al., 2019; Weber et al., 2022). G301D, D303H, and E525K (group#5), as well as L353P and G569R (group#3), were fully resistant to MCC950, either because they do not bind to MCC950, such as L353P (Vande Walle et al., 2019), or MCC950 is not sufficient to stabilize the inactive conformation. The group#3 mutants were activated upon priming, indicating that the mechanism of NLRP3 priming may not rely solely, or at all, on an inactive cage structure destabilization. Indeed, mutants activated by the activation signal nigericin without priming (group#2) were highly sensitive to MCC950 to a similar extent as the WT NLRP3, suggesting that these mutants form inactive structures and that the activation signal is at least involved in destabilizing this inactive form. Alternatively, MCC950 may inhibit NLRP3 activity independently of stabilizing the cage per se, but rather the closed conformation of the NACHT domain, e.g., when bound to NEK7, or additional other mechanisms.

While CY-09 is also considered a direct inhibitor of NLRP3, its action mechanism is poorly characterized (Jiang et al., 2017). It is supposed to compete with nucleotide binding to NLRP3; yet, a recent study has reported that CY-09 does not inhibit NLRP3 ATPase activity (Brinkschulte et al., 2022). Our results partly confirmed NLRP3 A352V (group#5) sensitivity to CY-09 (Jiang et al., 2017). In contrast to MCC950, no gain-of-function mutations showed full resistance to CY-09, consistent with CY-09 targeting residues indispensable for NLRP3 inflammasome assembly, suggesting that CY-09 and its derivatives may have broader activity than MCC950 for future clinical perspectives against NLRP3-AID. However, as a proposed ATP-competitive inhibitor, CY-09 might exhibit many off-target effects.

G5 is a deubiquitinase inhibitor that maintains NLRP3 in a ubiquitinated inactive form (Py et al., 2013). Consistent with the lack of specificity of G5 targeting multiple deubiquitinases, and the reports of multiple ubiquitination sites on NLRP3, none of the *NLRP3* gain-of-function mutants showed resistance to G5 (Py et al., 2013; Han et al., 2015; Tang et al., 2020; Wang et al., 2021). Among all mutants, NLRP3 A352V (group#5) and T436N (group#4) showed lower sensitivity to G5 upon treatment with doxycycline only and LPS only, respectively. However, as the amplitude of the activation was low in these conditions, the low percentage of inhibition may be artefactual and should be interpreted with caution. Noteworthy, constitutively active mutants were inhibited by G5, indicating that ubiquitination and deubiquitination may occur at least in part independently of priming and activation signals. The hypothesis that G5 may have an additional target downstream of NLRP3 in the pathway is ruled out by the specificity of G5 to inhibit the NLRP3 but neither the AIM2 nor the NLRC4 inflammasomes (Py et al., 2013).

CRT0066101 is a pan-PKD (protein kinase D) inhibitor targeting PKDs-mediated NLRP3 S293 phosphorylation upon activation signals that control the release of NLRP3 from an intracellular membrane compartment to the cytosol for the assembly of the inflammasome (Zhang et al., 2017). None of the group#2 and #4 mutants were inhibited by CRT0066101 when activated by activation signal in the absence of priming (Fig. 4, Dox+Nig only), indicating that in this context NLRP3 activation occurs independently of PKDs. Further investigation would be necessary to test if these mutants are released from the intracellular membranes independently of PKDs, display activity while associated with the membranes, or if they are not recruited to the membranes. In the latter hypothesis, priming might control NLRP3 recruitment to intracellular membranes or may disrupt an alternative pathway that bypasses the requirement for PKD by activating another kinase or another mechanism to control NLRP3 release from the intracellular membrane. Constitutive activity of most of the group#5 mutants was sensitive to CRT0066101, indicating that basal PKD activity may be required.

Altogether, our study constitutes the most comprehensive comparative analysis of *NLRP3* mutations associated with autoinflammation reported so far. Our results reveal the functional diversity of *NLRP3* gain-of-function mutations that should be taken into consideration for atypical NLRP3-AID diagnosis and specific targeted treatment perspectives.

## Materials and methods

### Study approval

The study was carried out in the setting of the ENFLAMAI protocol, which was previously approved by the French Comité de Protection des Personnes (#L16-189) and by the French Comité Consultatif sur le Traitement de l’Information en matière de Recherche dans le domaine de la Santé (#16.864). The experiments conformed to the principles set out in the World Medical Association Declaration of Helsinki and the Department of Health and Human Services’ Belmont Report. Anonymous healthy donors’ blood was provided by the Etablissement Français du Sang (EFS) in the framework of convention #14-1820 between Inserm and EFS. Informed consent was received from participants prior to inclusion in the study.

### NLRP3 variants nomenclature

All NLRP3 amino acids are numbered according to the Infevers database European nomenclature (https://infevers.umai-montpellier.fr/web/) (Milhavet et al., 2008). Their pathogenicity was classified by an international consortium of expert geneticists as previously described (Van Gijn et al., 2018). The exons (Table 1) are labeled according to the current NCBI NM_001243133.2 (including one upstream exon compared with the previous NM_001243133.1 regularly used in diagnosis analysis).

### Cell culture

Lenti-X 293T cells (Takara) were cultured at 0.5–1 × 10^6^ cells/ml in Dulbecco’s modified Eagle’s Medium (DMEM) GlutaMax-I supplemented with 1X penicillin/streptomycin (PS) and 10% fetal bovine serum (FBS) (Gibco). NLRP3-deficient U937 cells have been previously described (Lagrange et al., 2018). U937 and reconstituted U937 NLRP3-deficient cells were cultured at 0.25–1 × 10^6^ cells/ml in Roswell Park Memorial Institute (RPMI) 1640 GlutaMax-I supplemented with 1X PS and 10% FBS (Gibco). Monocytes were purified from 10 to 20 ml of blood samples (drawn the day before in heparin tubes) using EasySep Direct Human Monocyte Isolation kit (StemCell) and cultured at 0.07–0.15 × 10^6^ cells/ml in RPMI-1640 GlutaMax-I supplemented with 1X PS and 10% FBS (Gibco). The purity of the monocyte fractions was assessed by anti-CD45-APCR700 and anti-CD14-PE-CF594 (BD Horizon) staining and FACS analysis, and ranged between 66 and 82% CD45+/CD14+ (gated on total cells) and 81–95% (gated on CD45%).

### Plasmids

Human *NLRP3* were cloned in pENTR1A (Invitrogen) from Flag-NLRP3 encoding plasmids kindly shared by F. Martinon (University of Lausanne, Lausanne, Switzerland). Mutations were performed using QuickChange II kit (Agilent Technologies). cDNAs were transferred in pInducer21 using recombination Gateway LR clonase Enzyme mix kit (Thermo Fisher Scientific) (Meerbrey et al., 2011). pMD2.G and pCMVR8.74 were gifts from D. Trono (plasmid #12259 and #22036; Addgene).

### Reagents

The following reagents were used: polyethylenimine (PEI; Sigma-Aldrich), polybrene (Santa-Cruz), PMA (Invivogen), doxycycline (Sigma-Aldrich), LPS (O111:B4; Sigma-Aldrich), nigericin (Invivogen), ATP (Sigma-Aldrich), MCC950 (Adipogen), CY-09 (Tocris), G5 (Calbiochem), CRT0066101 (Tocris), PI (Immunochemistry Technologies), Hoechst (Immunochemistry Technologies).

### Lentivector preparation and cell transduction

1.6 × 10^6^ Lenti-X 293T cells were cotransfected with pMD2.G (10 μg), pCMVR8.74 (30 μg), and pInducer21-NLRP3 or its variants (40 μg) using PEI to produce lentivectors. Lentivectors were concentrated by ultracentrifugation (30,000 rpm, 1 h) and used to transduce 1.5 × 10^5^ U937 or NLRP3-deficient U937 cells in the presence of polybrene (8 μg/ml) (Lagrange et al., 2018). 4 days later, transduced GFP-positive cells were sorted by cytometry as bulk.

### Western blot (WB) analysis

To assess NLRP3 protein levels, all U937 cells were differentiated with PMA (50 ng/ml, 16 h) and treated with LPS (40 ng/ml, 3 h) or doxycycline (0.5–2 μg/ml, 3 h). Cells were directly lysed in sample buffer 2X. Samples were analyzed by SDS-PAGE and transferred to polyvinylidene difluoride membranes. The following antibodies were used: anti-NLRP3 (Cryo2; Adipogen), anti-IL-1β (D3U3E; Cell Signaling), anti-IL-18 (D2F3B; Cell Signaling), anti-LaminB (B-10; Santa Cruz Biotechnology), anti-actin (C4; Sigma-Aldrich), HRP-anti-Mouse IgG (H+L) (Promega).

### Cell death assay

To assess U937 permeabilization, 0.3 × 10^6^ cells/ml were plated in media without phenol red. The next day, cells were treated with doxycycline (1 μg/ml, 3 h), LPS (40 ng/ml, 2 h), and nigericin (15 μg/ml) before time-lapse imaging using CQ1 high-content screening microscope (Yokogawa). In the inhibition assay, MCC950 (1 μM), CY-09 (50 μM), G5 (1 μM), CRT0066101 (0.5 μM), or DMSO vehicle (1% final in all conditions) were added 20 min before doxycycline, LPS, or nigericin as indicated. To assess monocyte permeabilization, 0.07–0.15 × 10^6^ cells/ml were plated in media without phenol red and directly treated with MCC950 (1 μM, 3h 15), LPS (40 ng/ml, 3 h), and nigericin (5 μg/ml) before time-lapse imaging using CQ1 high content screening microscope (Yokogawa). PI (1.25 μg/ml) and Hoechst (0.2 μg/ml) were added 2 h before imaging. Two images/well were taken every 15 min for 2 h using 10× objectives (UPLSAPO 10X/0.4). Images were quantified using the CQ1 software (Yokogawa). Briefly, the analysis was based on the “Total count in individual object” analysis module, which consists of reducing noise (MeanImage, Mask size 1 μm), thresholding the image (threshold gray level 150), removing pixels from the edges (OpeningCircle, 5 μm), separating adjacent nuclei (FindMaximumDistance, with Minimum Point Distance 4 μm and Remove Size 0.1 μm, then DilationCircle 3 μm, and DivideEachRegion), integrating these last results with the intensity thresholding results (ExpandRegion3D) and applying a final filter size (50–500 μm). LDH release was assessed using Cytotoxicity Detection Kit^PLUS^ (Roche) according to the manufacturer’s instructions.

### Cytokine secretion assay

To assess IL-18, IL-1β, and TNF secretion, 0.25 × 10^6^ reconstituted U937 cells/ml were differentiated with PMA (50 ng/ml, 16 h). The next day, cells were treated with doxycycline (1 μg/ml, 4 h), LPS (40 ng/ml, 3 h), and nigericin (15 μg/ml, 1 h). Cell-free media were then analyzed using IL-18, IL-1β, and TNF ELISA kits (RnD Systems).

### Statistical analysis

The cell death was observed at 10 time points ranging from 0 to 135 min by 15-min intervals for each of the 34 *NLRP3* variants and *NLRP3* WT undergoing five treatments (i.e., untreated, Dox, Dox+LPS, Dox+Nig, and Dox+LPS+Nig), and studied as a proportion of the whole cell population.

Before performing the statistical analysis, the database was divided into 50 data subsets (5 treatments * 10 time points). To determine whether the proportion of cell death (response variable) in the NLRP3 variants (explanatory variable) deviates significantly from the proportion observed for the WT (reference), we fitted for each subset a generalized linear mixed model (glmm), beta family with logit link producing 50 results for each variant. The data subsets and the R code are provided in Table S1. In our model, the *NLRP3* variant type was set as the explanatory variable with the WT as the reference. The outcome variable in the model was the frequency of dead cells bounded in [0,1]. glmm can, similar to linear mixed-effects model (lmm), correct for the technical batch effect induced by the individual experiment. The P values obtained were corrected by the Bonferroni approach to take into account results for the five treatments. We used the R statistical environment, version 4.1.3, with the glmmTMB function from the glmmTMB package. We extracted from each model the regression coefficients and the P value for all NLRP3 variants compared with the WT and used the regression coefficients and the P values to construct the heatmaps. Results for Dox, Dox+LPS, and Dox+Nig treatments are shown. For visualization purposes, cut-offs were fixed to regression coefficients 1.5/−1.5 for Dox and Dox+LPS, and 0.5/−0.5 for Dox+Nig, with P value<0.05. For unsupervised clustering, the initial AUC dataset was divided into five data subsets (5 treatments). For each data subsets, we fitted a linear mixed model (GLMM) R programming language, yielding five models. The data subsets and the R code are provided in Table S2.

IL-1β, IL-18, and TNF concentrations were measured for the 34 *NLRP3* variants and *NLRP3* WT upon three treatments (i.e., Dox, Dox+LPS, and Dox+Nig). One dataset per cytokine * treatment was created, and a lmm was realized for each dataset to assess the dependence of the cytokine concentration on the variant. Each dataset comprised 536–564 observations. The data subsets and the R code are provided in Table S3. The outcome in the model was the log1p(log1p(X)=log(1+X)) of the cytokine concentration given in pg/ml while the right side of the model was the *NLRP3* variant. The *NLRP3* WT was set as the reference. lmm was appropriate due to its ability to correct for the batch effect triggered by the individual experiment. We used the R statistical environment, version 4.1.3 with the lme function from the nlme package. We extracted from each model the regression coefficients and the P value for all NLRP3 variants compared with the WT and used the regression coefficients and the P values to construct the heatmaps. For visualization purposes, cut-offs were fixed to regression coefficients 1/−1 with P value <0.05.

The effect of the NLRP3 inhibitors was tested by comparing AUC obtained in the presence of each of the four inhibitors (i.e., MCC950, CY-09, G5, and CRT0066101) to the AUC obtained without inhibitor (vehicle only, used as the reference state). Of note, inhibitors were tested on the WT NLRP3 only upon Dox+LPS+Nig treatment. The database was divided into 137 data subsets (34 genotypes [NLRP3 variants] * 4 treatments [Dox, Dox+LPS, Dox+Nig, Dox+LPS+Nig] + 1 genotype [NLRP3 WT] * 1 treatment [Dox+LPS+Nig]). In each dataset, we transformed the AUC to the logarithmic scale. An lmm was fitted for each genotype undergoing a given treatment (corresponding to each data subset). The data subsets and the R code are provided in Table S4. The model response variable was log10(AUC) and the explanatory variable was the inhibitors. As data diagnosis proved a lawful steady batch effect of the independent experiment, lmm modeling was used to correct for this batch effect technical bias in the data. The P values obtained were corrected by the Bonferroni approach to take into account results for the four treatments (Dox, Dox+LPS, Dox+Nig, and Dox+LPS+Nig). We extracted from each model the regression coefficients and the P values for all inhibitors compared with the “no inhibitor” level to construct the heatmaps. For visualization purpose, cut-offs were fixed to regression coefficients less than −0.1 with P value <0.05. Regression coefficient = log10(Fold change). We used the R statistical environment, version 4.1.3, with the lme function from the nlme package.

### Structural analyses

For the evaluation of CAPS disease mutations, the decameric structures of inactive human NLRP3 bound to ADP and MCC950 (7PZC) (Hochheiser et al., 2022) and of active human NLRP3 bound to ATPγS and NEK7 (8EJ4) (Xiao et al., 2023) were used. Interaction analysis of the mutant sites in the inactive and active states was performed using PDBePISA (Krissinel and Henrick, 2007). The effect of disease mutations on the activation mechanism through rotation of the FISNA-NBD-HD1 subdomains relative to the WHD-HD2-trLRR-cnLRR and the binding to NEK7 was performed visually and using PDBePISA. Molecular diagrams were drawn using the PyMOL molecular graphics suite (https://pymol.org/2/).

### Online supplemental material

Fig. S1 shows NLRP3 expression in reconstituted NLRP3-deficient U937 cell lines. Fig. S2 shows pyroptosis in reconstituted U937 cells upon NLRP3 expression, priming, and/or activation signals. Fig. S3 shows IL-18, IL-1β, and TNF secretion in reconstituted U937 cells upon NLRP3 expression, priming, and/or activation signals. Fig. S4 shows statistical analysis of IL-18, IL-1b, and TNF secretion. Fig. S5 shows the sensitivity of *NLRP3* variants to NLRP3 inhibitors (group#5 and #4). Fig. S6 shows the sensitivity of NLRP3 variants to NLRP3 inhibitors (group#3–1). Fig. S7 shows details of the disease-causing mutant sites in NLRP3 and MCC950 inhibition. Table S1 shows data subsets corresponding to the cell death kinetics analysis. Table S2 shows data subsets corresponding to the cell death AUC analysis used for unsupervised clustering. Table S3 shows data subsets corresponding to the cytokine secretion analysis. Table S4 shows data subsets corresponding to the sensitivity to NLRP3 inhibitors analysis used in this study.

## Supplementary Material

Table S1 shows data subsets corresponding to the cell death kinetics analysis.

Table S2 shows data subsets corresponding to the cell death AUC analysis used for unsupervised clustering.

Table S3 shows data subsets corresponding to the cytokine secretion analysis.

Table S4 shows data subsets corresponding to the sensitivity to NLRP3 inhibitors analysis.

SourceData F3 is the source file for Fig. 3.

SourceData FS1 is the source file for Fig. S1.

SourceData FS2 is the source file for Fig. S2.

## Data Availability

Data are available in the article itself and its supplementary materials.
