# Supplementary figures and images for: Functional diversity of NLRP3 gain-of-function mutants associated with CAPS autoinflammation
Source: J Exp Med. 2024 Mar 26;221(5):e20231200. doi: 10.1084/jem.20231200 (PMC10966137; doi:10.1084/jem.20231200)

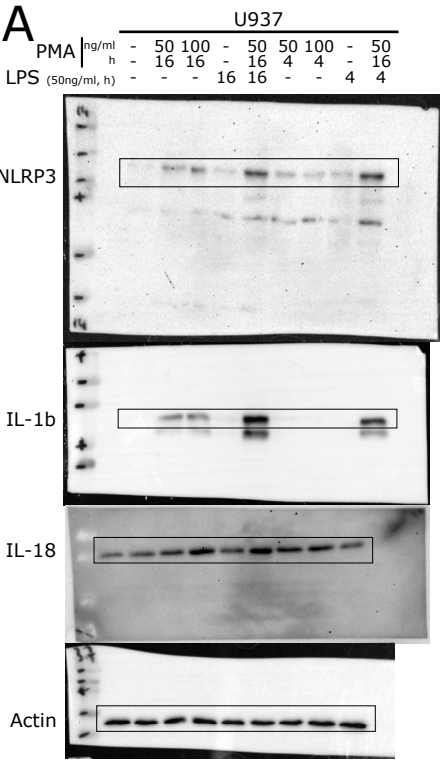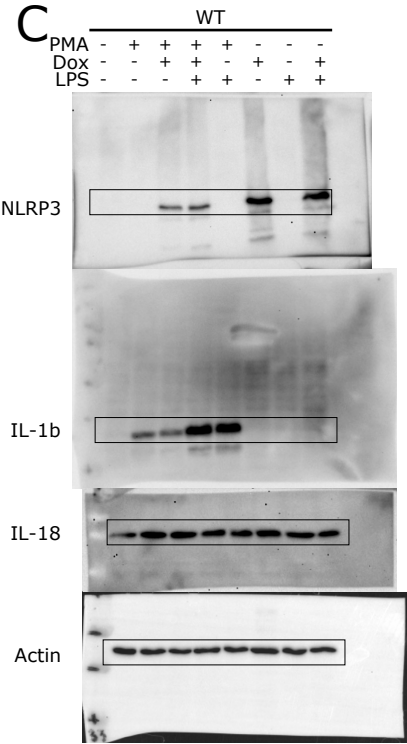

Supplement: SourceData F3 — is the source file for Fig. 3. [file JEM_20231200_SourceDataF3.pdf]

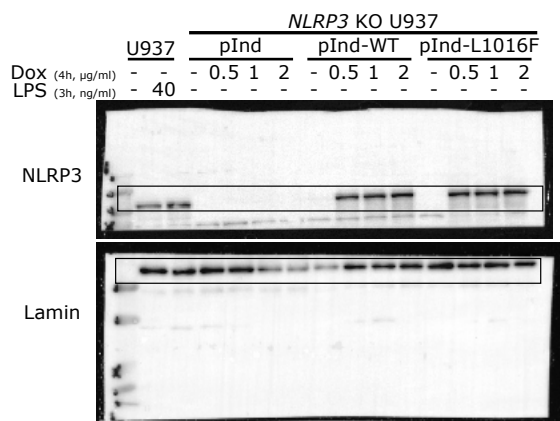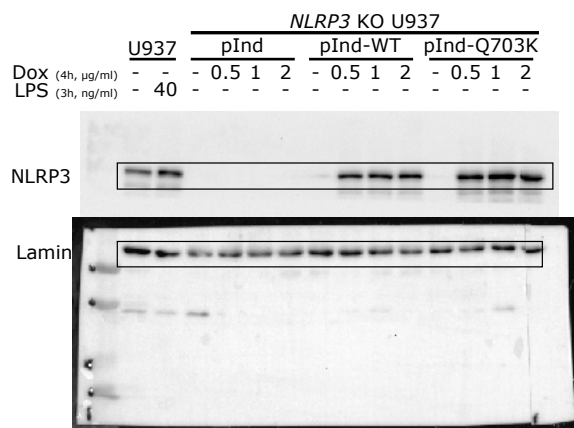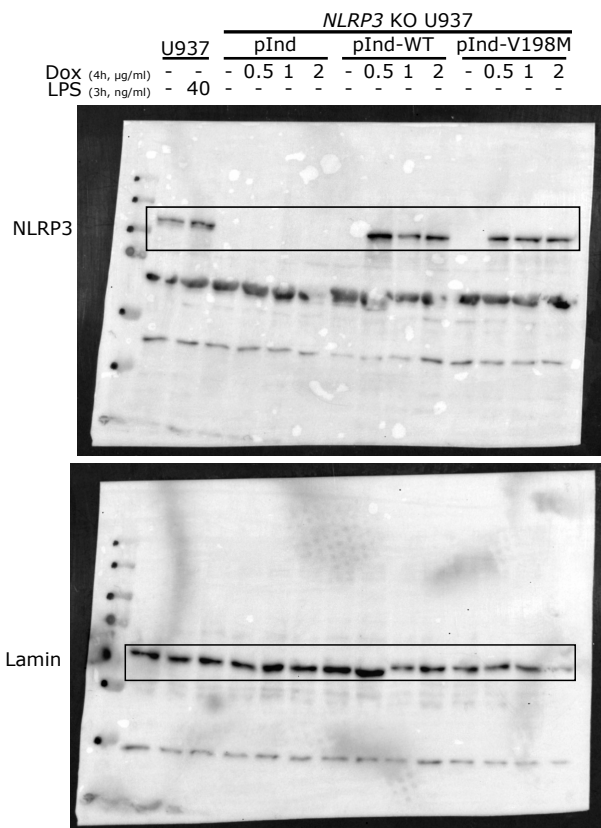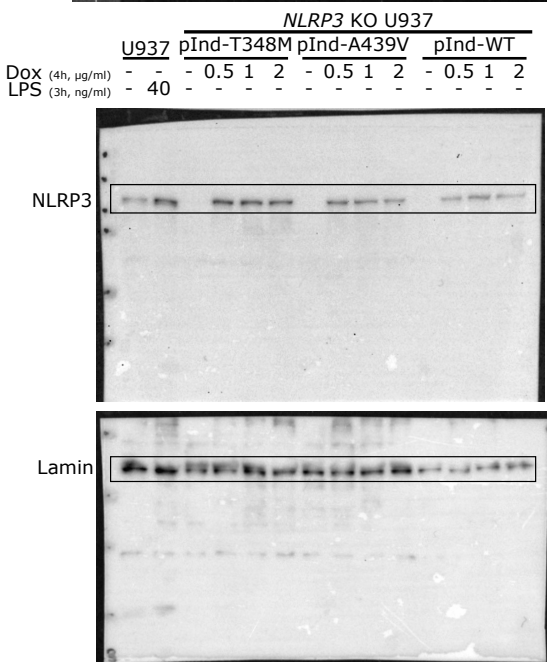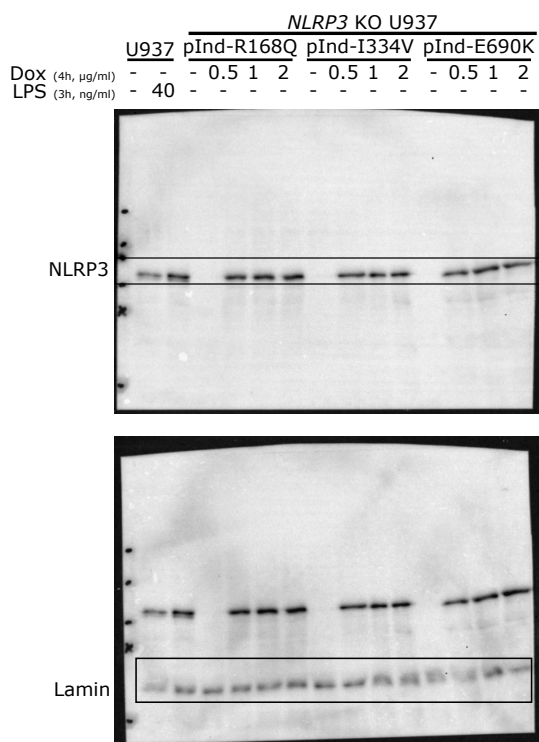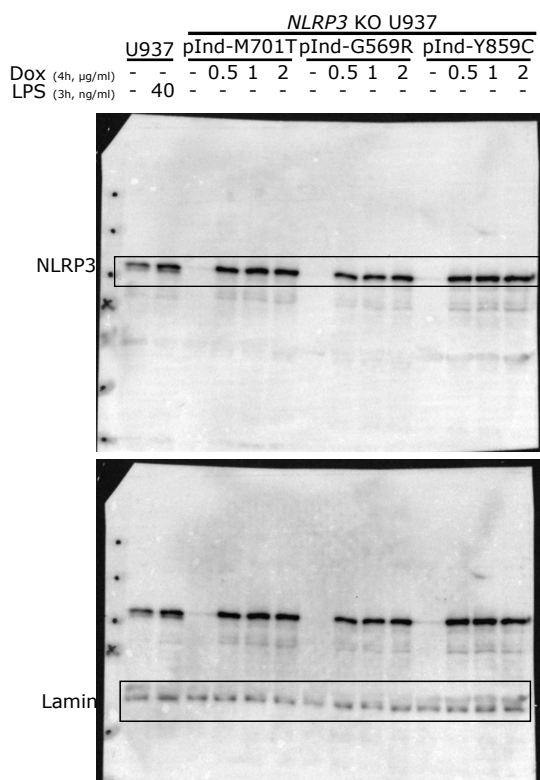

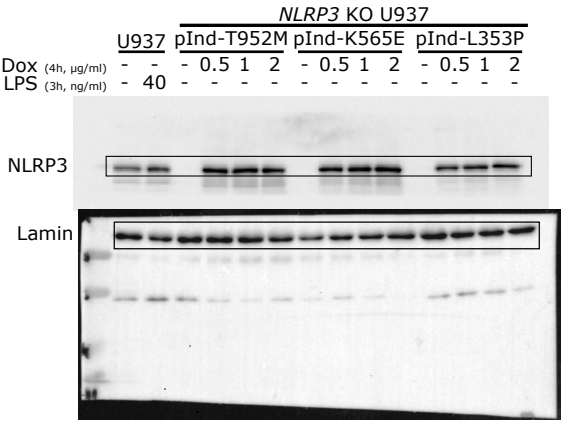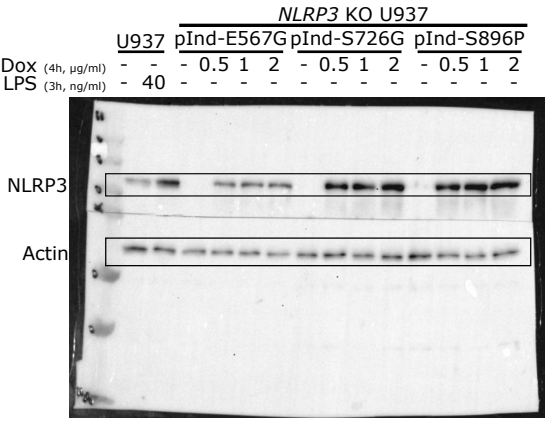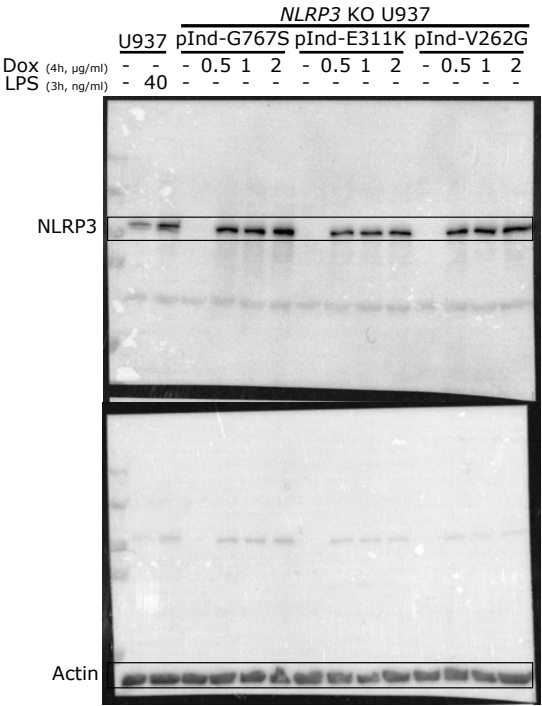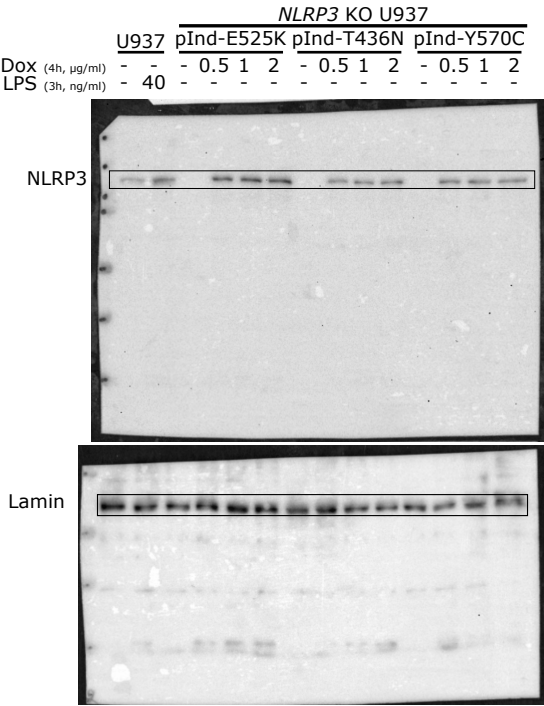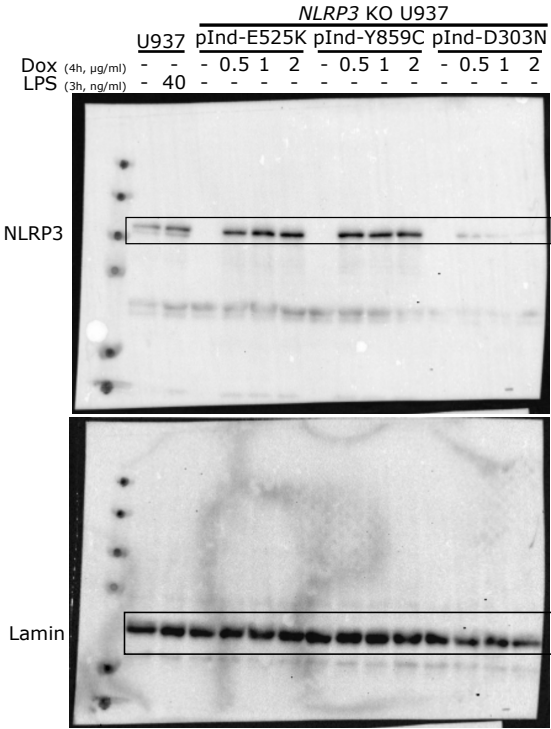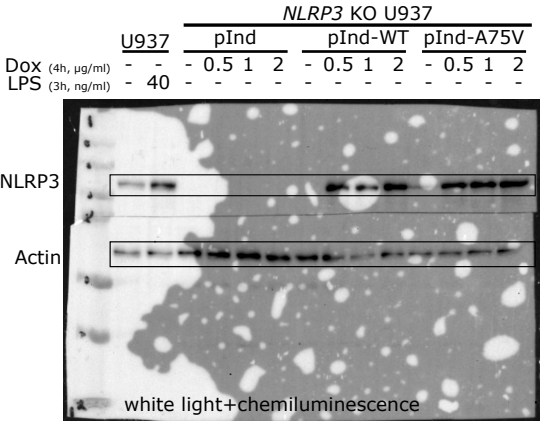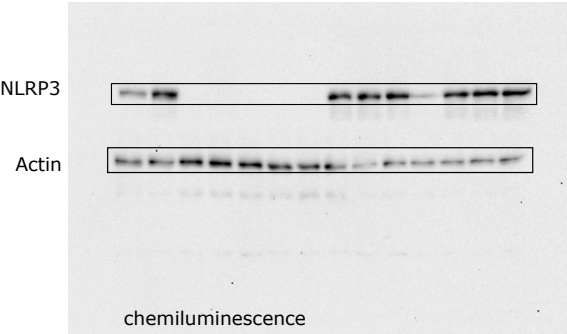

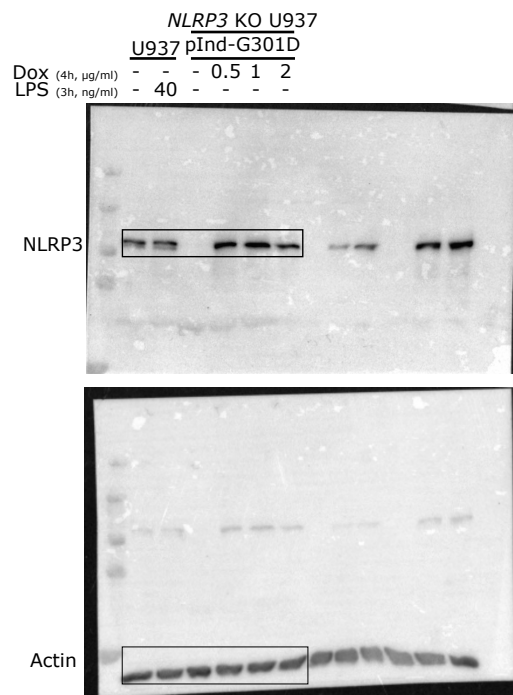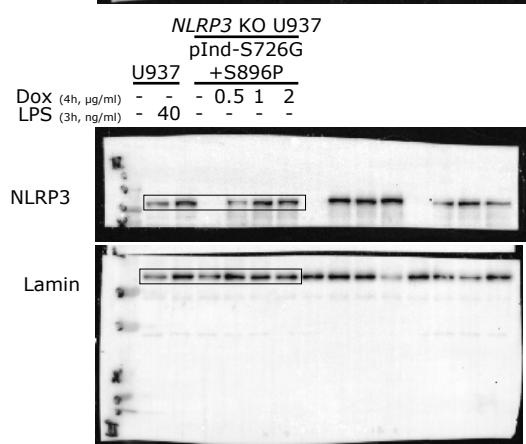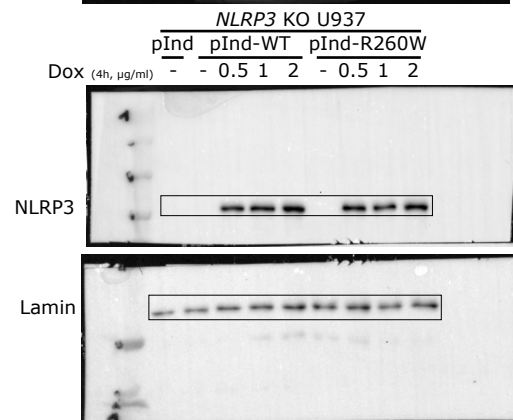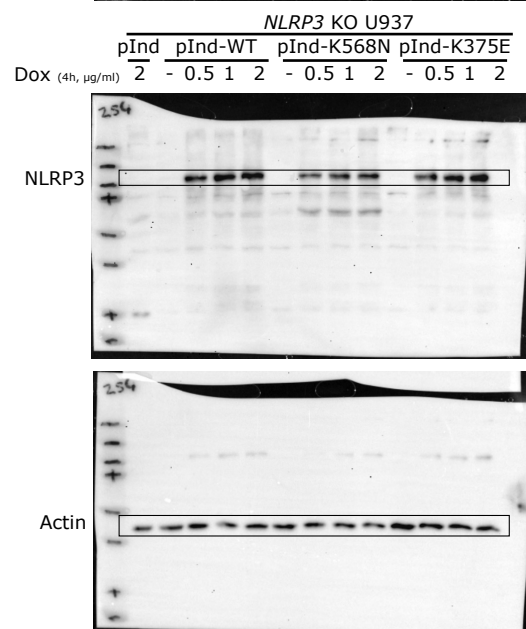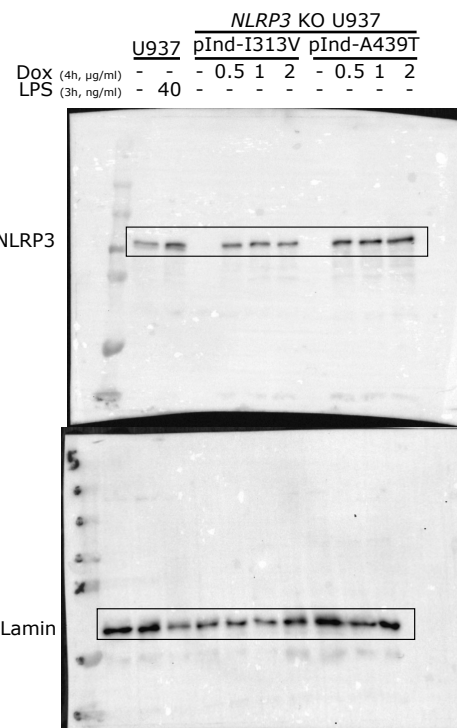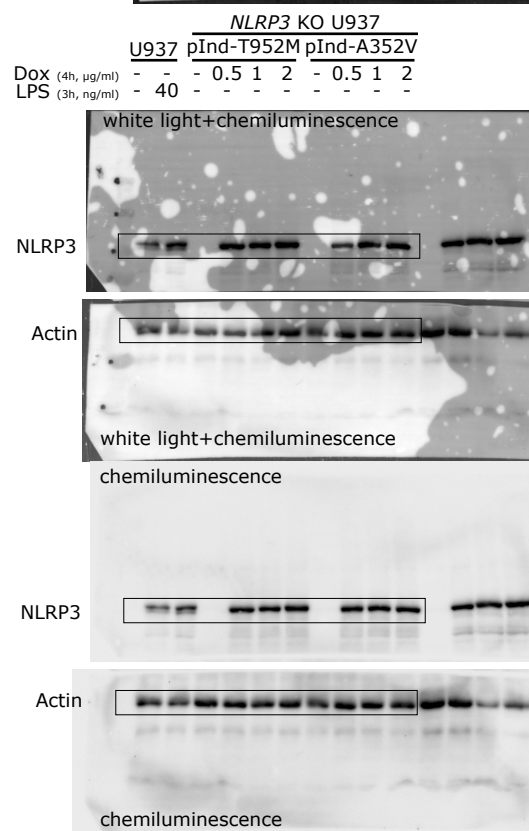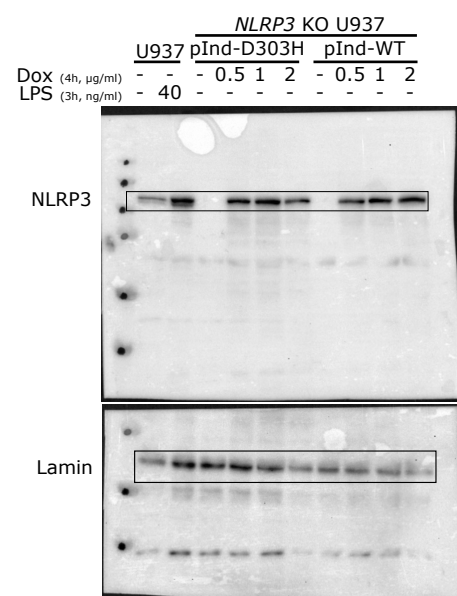

Supplement: SourceData FS1 — is the source file for Fig. S1. [file JEM_20231200_SourceDataFS1.pdf]

H

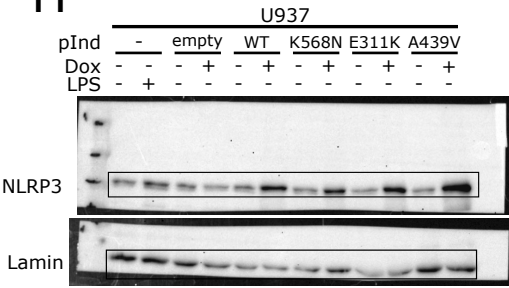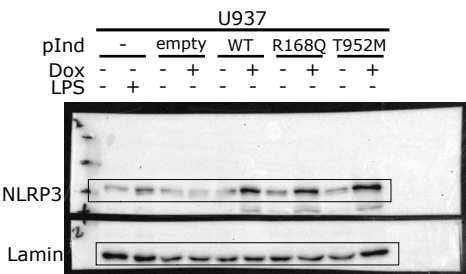

SourceDataFS2

Supplement: SourceData FS2 — is the source file for Fig. S2. [file JEM_20231200_SourceDataFS2.pdf]
